# Modulation of innate immunity related genes resulting in prophylactic antimicrobial and antiviral properties

**DOI:** 10.1186/s12967-024-05378-2

**Published:** 2024-06-17

**Authors:** Veronica Ferrucci, Marco Miceli, Chiara Pagliuca, Orazio Bianco, Luigi Castaldo, Luana Izzo, Marica Cozzolino, Carla Zannella, Franca Oglio, Antonio Polcaro, Antonio Randazzo, Roberta Colicchio, Massimiliano Galdiero, Roberto Berni Canani, Paola Salvatore, Massimo Zollo

**Affiliations:** 1 Department of Molecular Medicine and Medical Biotechnology (DMMBM), University of Naples ‘Federico II’, Via Sergio Pansini 5, 80131 Naples, Italy; 2 CEINGE Biotecnologie Avanzate ‘Franco Salvatore’, Via Gaetano Salvatore 486, 80145 Naples, Italy; 3 Elysium Cell Bio Ita, Via Gaetano Salvatore 486, 80145 Naples, Italy; 4 Department of Pharmacy, University of Naples ‘Federico II’, Via Domenico Montesano 49, 80131 Naples, Italy; 5 Dipartimento Di Scienze Mediche Traslazionali, University of Naples Federico II, Via Sergio Pansini 5, 80131 Naples, Italy; 6 Department of Experimental Medicine, University of Campania “Luigi Vanvitelli”, 80138 Naples, Italy; 7 Polcaro Fitopreparazioni S.R.L, Via Sant Agnello, 9 D; 80030, Roccarainola, Naples, Italy; 8 UOC of Virology and Microbiology, University Hospital of Campania “Luigi Vanvitelli”, 80138 Naples, Italy; 9 DAI Medicina di Laboratorio e Trasfusionale, University of Naples Federico II, Via Sergio Pansini 5, 80131 Naples, Italy

**Keywords:** Co-infections, COVID-19, SARS-CoV-2, Sincytial respiratory virus, Influenza virus, Innate immunity, Nutraceutical formula, Polyphosphates

## Abstract

**Background:**

The innate immunity acts during the early phases of infection and its failure in response to a multilayer network of co-infections is cause of immune system dysregulation. Epidemiological SARS-CoV-2 infections data, show that Influenza Virus (FLU-A-B-C) and Respiratory Syncytial Virus (RSV) are co-habiting those respiratory traits. These viruses, especially in children (mostly affected by ‘multi-system inflammatory syndrome in children’ [MIS-C] and the winter pandemic FLU), in the aged population, and in ‘fragile’ patients are causing alteration in immune response. Then, bacterial and fungal pathogens are also co-habiting the upper respiratory traits (*e.g., Staphylococcus aureus* and *Candida albicans*), thus contributing to morbidity in those COVID-19 affected patients.

**Methods:**

Liquid chromatography coupled with high-resolution mass spectrometry using the quadrupole orbital ion trap analyser (*i.e.,* UHPLC-Q-Orbitrap HRMS) was adopted to measure the polyphenols content of a new nutraceutical formula (Solution-3). Viral infections with SARS-CoV-2 (EG.5), FLU-A and RSV-A viruses (as performed in BLS3 authorised laboratory) and real time RT-PCR (qPCR) assay were used to test the antiviral action of the nutraceutical formula. Dilution susceptibility tests have been used to estimate the minimum inhibitory and bactericidal concentration (MIC and MBC, respectively) of Solution-3 on a variety of microorganisms belonging to Gram positive/ negative bacteria and fungi. Transcriptomic data analyses and functional genomics (*i.e.,* RNAseq and data mining), coupled to qPCR and ELISA assays have been used to investigate the mechanisms of action of the nutraceutical formula on those processes involved in innate immune response.

**Results:**

Here, we have tested the combination of natural products containing higher amounts of polyphenols (*i.e.,* propolis, *Verbascum thapsus* L., and *Thymus vulgaris* L.), together with the inorganic long chain polyphosphates ‘polyPs’ with antiviral, antibacterial, and antifungal behaviours, against SARS-CoV-2, FLU-A, RSV-A, Gram positive/ negative bacteria and fungi (*i.e., Candida albicans*). These components synergistically exert an immunomodulatory action by enhancing those processes involved in innate immune response (*e.g.,* cytokines: IFNγ, TNFα, IL-10, IL-6/12; chemokines: CXCL1; antimicrobial peptides: HBD-2, LL-37; complement system: C3).

**Conclusion:**

The prophylactic antimicrobial success of this nutraceutical formula against SARS-CoV-2, FLU-A and RSV-A viruses, together with the common bacteria and fungi co-infections as present in human oral cavity, is expected to be valuable.

**Supplementary Information:**

The online version contains supplementary material available at 10.1186/s12967-024-05378-2.

## Introduction

Respiratory illnesses due to infectious diseases have re-emerged in unforeseeable patterns after the pandemic. Indeed, as of 6th January 2024, the morbidity and mortality weekly report (MMWR) by the Centers for Disease Control and Prevention (CDC) (https://www.cdc.gov/ncird/surveillance/respiratory-illnesses/index.html) reported n.76.574 ‘coronavirus disease 2019’ (COVID-19), n.106.331 influenza (FLU) and n.19.748 respiratory syncytial virus (RSV) reported cases, with n.202.626 co-infected patients. This epidemiological picture underpins that the microbial co-infections retain their crucial role also in post-pandemics era.

Severe Acute Respiratory Syndrome Coronavirus 2 (SARS-CoV-2), a pathogen positive-sense single-stranded RNA virus, is the etiopathological agent of the pandemic COVID-19 responsible for substantial morbidity and mortality worldwide [1]. Respiratory droplets and aerosols are both causes of SARS-CoV-2 transmission with a median incubation period of 4.5 days before symptoms onsets [2]. Mild-to moderate COVID-19 patients experience cough, fever, self-reported olfactory and taste disorders, and gut microbiome dysbiosis [2]. In contrast, the severe illness begins 1 week after symptom onset with dyspnoea and proceeds with a progressive respiratory failure (‘acute respiratory distress syndrome’, ARDS), systemic hyperinflammation, and extrapulmonary disease (*e.g.,* cardiac, kidney and liver injury, coagulopathy and shock) [2]. Furthermore, COVID-19 patients often present persistent symptoms after infection, referred to as ‘long COVID’, occurring in at least 10% of mild and severe SARS-CoV-2-infected people worldwide [3].

The microbial co-infection from virus, bacteria and fungi exacerbates the difficulties of treatment and prognosis of COVID-19 patients [4]. Indeed, the bacterial ‘community-acquired pneumonia’ (CAP) co-infection confers a greater risk of ‘in-hospital mortality’ as compared to the other risk factors, such as advanced age and comorbidities [5]. In this regard, a shift in the prevalence of respiratory pathogens in CAP occurred during COVID-19 pandemic [6]. *Staphylococcus aureus* is the most frequent Gram-positive pathogen affecting the respiratory tract found in SARS-CoV-2 co-infections [5, 6]. Furthermore, changes of intestinal microbiota have been also reported in COVID-19 patients that showed reduction in bacteria diversity with increased opportunistic pathogens, such as *Streptococcus*, *Rothia*, *Escherichia coli* and *Shigella* and reduction of beneficial symbionts [7]. Regarding the other classes of etiological agents, Influenza virus and RSV are predominantly found among those respiratory viruses contributing to the co-infection with SARS-CoV-2 [4, 8, 9], especially among children aged 0–5 years [10]; While *Candida albicans* is the most common coinfect fungus in COVID-19 patients [11]. Altogether these results suggest that COVID-19 infection increases the host susceptibility to other pathogen co-infections, probably via inducing long-lasting changes in innate- and adaptive- immune functions in both adults and children [3, 12, 13].

COVID-19 disease is generally accomplished by immune dysregulation in SARS-CoV-2-infected host that is firstly characterized by ‘immunosuppression’ [14] and later followed by ‘hyperinflammation’ [15]. The hyperinflammatory status is mediated by pro-inflammatory cytokines (*i.e.,* ‘cytokine storm’), triggered by inflammatory signaling activation (e.g., nuclear factor kappa-light-chain-enhancer of activated B cells, NF-kB [16]) that are responsible for immune cells dysfunctionalities (*e.g.,* lymphopenia), thus increasing the susceptibility to co-infections by other microorganisms, including viruses, bacteria, and fungi [17].

Of importance, a crucial role during the early phases of infection is made by the antiviral innate immune response, including complement systems, interferons (IFN), chemokines, and immune cells (*e.g.,* macrophages) whose role mainly consist in limiting the viral propagation via modulating cytokine production and, generally 2–3 weeks later after contact with the virus, inducing the adaptive immune response [18]. Thus, a failure of innate immunity may result in an abnormal acquired immune host response that causes those critical COVID-19 conditions.

Therefore, two different therapeutic time windows must be considered in the clinical management of the immunopathological viral response. For the early phases of infection or also ‘prophylaxis’, immunostimulant agents could be helpful to enhance the innate immunity against microbial infections. In contrast, once the virus has overcome the initiation of infection and the ‘active’ viral replicative stage has started, the systemic treatments should be aimed at inhibiting the hyperinflammation.

To date, only vaccines have been the most effective prophylactic treatment against COVID-19 pandemic. Nevertheless, the existing anti-Spike antibodies show a weakly activity or inactiveness against the new sub-lineages of the Variants of Concern (VOC) Omicron, such as BA.1, BA.1.1, BA.2, BA.4 and BA.5 [19, 20], including the latest emergent XBB: EG.5.1 and XBB.2.3 [21]. Other COVID-19 therapeutics include antivirals, antithrombotic, therapies for respiratory failure, neutralizing antibodies, modulators of the renin–angiotensin–aldosterone system, vitamins, immunomodulatory agents (IMIDs, *e.g.,* glucocorticoids and cytokine antagonists) and anti-inflammatory drugs (AIDs) [19]. Thus, the development of effective treatment and prophylactic strategies against SARS-CoV-2 variants still represent an unmet medical need.

In the medical management of patients affected by COVID-19, some valuable therapeutic strategies imply the use of AIDs, especially those non-steroidal AIDs (NSAIDs), to mitigate the ongoing excessive inflammatory response occurring after the early phases of the disease [22]. Indeed, several observational studies have indicated that the anti-inflammatory therapy with NSAIDs (*e.g.,* Aspirin, Ibuprofen, and Indometacin), and especially the use of selective COX-2 inhibitors (*e.g.,* Celecoxib and Nimesulide), can be safely used among patients positive to SARS-CoV-2 [23] showing beneficial effectiveness for the outpatient treatment of early COVID-19 symptoms [22], thus giving a ‘protection’ against the progression of the disease towards a severe illness. Nevertheless, the therapy with NSAIDs should be avoided in those patients younger than 12 years and during pregnancy, due their possible multiple adverse effects, including gastrointestinal bleeding, cardiovascular, nephrotoxicity, especially for people older than 65 years [24].

Nutraceuticals are potential alternatives to NSAIDs for the management of various inflammatory diseases, including mild to moderate COVID-19 [25]. Among the plant-based nutraceuticals, polyphenols (which are grouped in four classes: phenolic acids, flavonoids, stilbenes and lignans) have received particular interest because of their key roles in a variety of biological activities, including anti-inflammatory and immunomodulatory functions [26]. These functionalities exerted by polyphenols are mainly due to modulation of NF-kB pathway that, in turn, affects inflammatory cytokines production and, therefore, immune cells activities [26]. Indeed, NF-kB has been already reported with several functions in modulating the innate immune response by promoting the M1-polarization of macrophages, maturation of dendritic cells and the recruitment neutrophils. While, in the context of the adaptive immunity, NF-kB has been found to activate B cells by enhancing their proliferation, maturation, and mature selection, and to drive the differentiation of CD4 T cells into different subtypes [27].

Propolis, a resinous complex formula generated by honeybees, has been reported with nutraceutical benefits due to its high amounts of polyphenols that are responsible for antibacterial, antiviral, anti-inflammatory, antioxidant and immunostimulant activities [28]. Indeed, propolis has been reported with antimicrobial function against human pathogenic viruses [29] (*e.g.,* Influenza FLU [30], Herpes simplex type-1 HSV-1 [30], RSV-1 [31], RSV-2 [32], human coronaviruses SARS-CoV-2 [30] and HCoV-OC43 [32], human adenovirus type 5 [32], human rhinovirus type 14 [32]) and bacteria (*e.g., Escherichia coli* [33] and *Pseudomonas aeruginosa* [29]), especially those belonging to Gram-positive class (including *Staphylococcus aureus* [29, 33, 34]).

*Thymus vulgaris* L. (species, *Plantarum*; family, *Lamiaceae*) is an herbaceous, perennial aromatic and medicinal plant, rich in bioactive compounds (including polyphenols) with anti-oxidant [35], antibacterial (*e.g.,* against *Escherichia coli* [35], *Pseudomonas aeruginosa* [35, 36], *Streptococcus salivarius* [36], *Streptococcus mutans* [36], *Streptococcus pyogenes* [36] and *Staphylococcus aureus* [36]), antifungal (*i.e., Candida albicans* [36]), antiviral (HSV [37], SARS-CoV-2 [38], human immunodeficiency virus HIV [39], and Influenza [39]), and anti-inflammatory functions [40].

*Verbascum thapsus* L. (species, *Plantarum*; family: *Scrophulariaceae*), is a monocarpic and biennial herb, and their flowers are a source of a wide variety of chemical constituents, including polyphenols [41]. *Verbascum thapsus* L. has been reported with antiviral efficiency (against pseudorabies virus [42], human coronavirus HCov-229E [43], HSV-1 [44] and influenza A [44]), antibacterial activity (especially against Gram positive strain, including *Staphylococcus aureus* [45]) with anti-inflammatory properties [46].

Here, we have developed a novel nutraceutical formula by combining propolis, *Thymus vulgaris* L. and *Verbascum thapsus* L. with the addition of long chain inorganic polyphosphates (polyPs). These latest are currently used as dietary additives (E452i, as approved by European Food Safety Authority [EFSA]) with low acute oral toxicity, absence of genotoxicity and carcinogenicity, and with an acceptable daily intake (ADI) for phosphates expressed as phosphorus of 40 mg/kg of body weight per day [47]. Inorganic polyPs are compounds comprised of chains of five to many hundreds of inorganic phosphate (Pi) residues that modulate a variety of biological processes, including cell metabolism [48], inflammation [49], chaperone-like functions [50] and neural transmission [51, 52]. Recently polyPs have been described with an antimicrobial activity as new therapeutics for chronic wounds in humans due to their properties to entrap bacteria [53]. Furthermore, polyPs were also found to act as cytoprotective agents against HIV-1 [54] and SARS-CoV-2 [55–59]. Indeed, prophylactic, and therapeutic treatment with polyPs resulted in inhibition of SARS-CoV-2 active replication in vitro. Their mechanisms of action involve *(i)* binding to Angiotensin Converting Enzyme 2 (ACE2) on the host cells and to viral RNA-dependent RNA polymerase (RdRp) in infected cells promoting their proteasome-mediated degradation, *(ii)* impairment of viral-host cell interaction through their binding to viral Spike (S) protein, and *(iii)* inhibition of inflammatory cytokines belonging to the cytokine storm via NF-kB modulation [55, 56]. Furthermore, the delivery of polyPs with a non-ambulatory nebulizer system also resulted in antiviral effects in vitro against SARS-CoV-2 [55].

Here, we show the antimicrobial effectiveness of a new nutraceutical formula named ‘Solution-3’ containing natural extracts from propolis, *Thymus vulgaris* L. leaves and *Verbascum thapsus* L. flowers, and polyPs (E452i).

We have here performed in vitro assays to verify the absence of cytotoxicity of the selected compounds alone and in combination by determining the half-maximal inhibitory concentration (IC_50_) on cell proliferation of human cells (*i.e.,* HEK-293 T) and by assessing the absence of apoptosis via caspase 3 enzymatic assays. We also showed that the nutraceutical formula possesses immunomodulatory properties that are mainly triggered by its high levels of polyphenols, as determined by chemically profiling the Solution-3 through liquid chromatography coupled with high-resolution mass spectrometry using the quadrupole orbital ion trap analyser (*i.e.,* UHPLC-Q-Orbitrap HRMS). Then, transcriptomic data analyses (*i.e.,* RNAseq), coupled to qPCR and ELISA assays, show modulation of both inflammatory cytokines and antimicrobial peptides (human beta defensin [HBD-2] and cathelicidin [LL-37]) with a crucial role in the innate immune response. Furthermore, we also have demonstrated a bacteriostatic and/ or bactericidal action of the nutraceutical formula against a variety of pathogens (gram-positive, gram-negative bacteria and *C. albicans*) that are mainly responsible for the upper respiratory trait infections and co-infections with SARS-CoV-2, thus determining the minimum inhibitory concentrations (MICs) and the minimum bactericidal concentrations (MBCs) of Solution-3. Finally, data obtained through in vitro experiments performed in a Biosafety Level 3 (BSL-3) authorized lab, have also shown the antiviral effectiveness of this nutraceutical formula against SARS-CoV-2, FLU-A and RSV-A2 in terms of viral propagation, including syncytia formation and inflammatory cytokines modulation.

Altogether, our data show the prophylactic and therapeutic benefits of a new nutraceutical in a nano-spray formulation that has the potential to boost the innate immune response during the first phases of infection, thus exerting antibacterial, antifungal, and antiviral efficacy against those pathogens responsible for those respiratory trait infections and co-infections occurring in post-pandemic era.

## Results

### A nutraceutical formula with high polyphenols content containing polyPs

PolyPs are currently used as dietary additives without presenting an appreciable risk to health showing an ADI for phosphates of 40 mg/kg of body weight per day [47]. Recently, medium (*i.e.,* polyP40) and long (*i.e.,* polyP120) chain length polyPs (in terms of Pi residues) have been reported with antiviral activity against SARS-CoV-2 [55, 56]. Furthermore, polyPs have received attention as possible therapeutics with some recent studies exploring their use in a several formulations (including nebulizer [55] and hydrogel [53]) for their antiviral [55–59], antibacterial [53] and immunomodulatory [55] activities.

We have here combined polyPs (*i.e.,* polymer with a wide range of chain length in terms of phosphate residues) with extracts from natural compounds aiming to develop a novel nutraceutical formula with antimicrobial potential. To this aim, we selected those ingredients already known to contain bioactive compounds (*i.e.,* polyphenols) with therapeutic benefits in terms of antiviral, antibacterial and/ or antifungal properties. Thus, we used extracts from propolis [29–34], common *Thymus vulgaris* L. (or thyme) leaves [35–39], and *Verbascum thapsus* L. (or mullein) flowers [42–45]. We used 0.8% NaCl in water as isotonic solvent for the production of the nutraceutical formula because of its potential use as nasal-spray formulation in the near future.

**Fig. 1:**
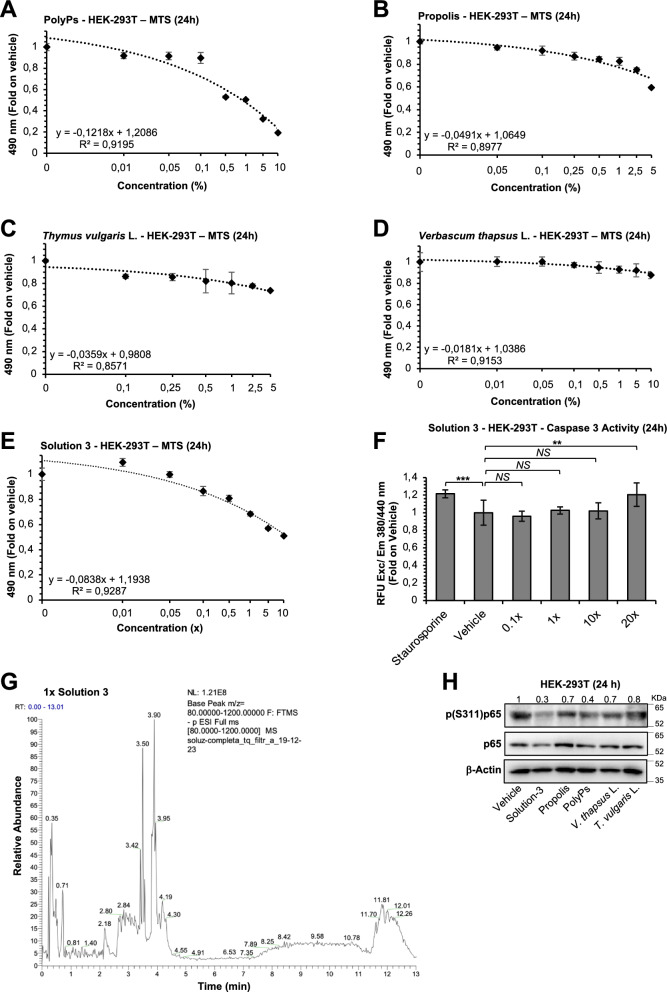
Solution-3 and its components with high polyphenol contents is not cytotoxic. **A**-**E** Graphs showing MTS assays performed in HEK-293 T cells (1 × 10^5^) treated for 24 h with polyPs (**A**, 0.01 to 10% μM), propolis (**B**, 0.05 to 5%), *Thymus vulgaris* L. leaves (Sp. Pl. 2: 591 [1753]; International Plant Names Index ‘IPNI’: Life Sciences Identifier ‘LSID’, urn:lsid:ipni.org:names:461765-1; species, *Plantarum*; collation: 2: 591; IPNI family, *Lamiaceae;* https://www.ipni.org/n/461765-1) (**C**, 0.1 to 5%), *Verbascum thapsus* L. flowers (Sp. Pl. [Linnaeus] 1: 177 [1753]; ‘IPNI’: ‘LSID’, urn:lsid:ipni.org:names:770183-1; species, *Plantarum*; collation, 1: 177; IPN family: *Scrophulariaceae*; https://www.ipni.org/n/770183-1) (**D**, 0.01 to 10%) or Solution-3 (**E,** 0.1 to 10x). Vehicle-treated cells (*i.e.,* 0.8% NaCl) were used as negative control. The absorbance values measured at 490 nm are shown as folds on vehicle-treated cells (see Additional file 1: Table S1). Values represent the average ± SD. N = 6 independent experiments per group. The regression equation, R-squared and IC_50_ values are reported. The IC_50_ is calculated through nonlinear regression analysis {[inhibitor] versus response (three parameters)} with ‘Quest Graph™ IC50 Calculator’ (AAT Bioquest, Inc., 8 Jan. 2024; https://www.aatbio.com/tools/ic50-calculator. The graphs are generated with Microsoft Excel (version 16.82). N = 6 independent experiments per group. R^2^, Coefficient of Determination. **(F)** Graph showing Caspase 3 assay in HEK-293 T cells (1.5 × 10^5^) treated with escalating doses of Solution-3 (0.1 to 20x) for 24 h. Treatments with 0.2 μM Staurosporine or with 0.8% NaCl were used as positive and negative control for the experiment, respectively. The relative fluorescent units (RFUs) recorded using a fluorescence reader with excitation at 380 nm and emission at 440 nm are shown as folds on vehicle-treated cells (Additional file 1: Table S2**)**. Values represent the average ± SD. **P < 0.01, ***P < 0.001 by unpaired two-tailed Student’s. The graph is generated with Microsoft Excel (version 16.82). N = 9 independent experiments per group. NS, not statistic. **(G)** Total Ion Chromatogram (TIC) of Solution-3 at 1 × concentration obtained through UHPLC Q-Orbitrap HRMS is shown. The relative abundance of polyphenols is shown on Y axis. The polyphenolic profile with the list of phenolic acids (benzoic and cinnamic acids) and flavonoids (flavonols, flavones, flavanones, isoflavones) are shown in Table 1 as μg/g values. (**H**) Representative immunoblotting analysis (with antibodies against the indicated proteins) of total cell lysates from HEK-293 T cells treated for 24 h with Solution-3 (0.01x), or with its components alone (*i.e.,* 0.625% propolis, 0.125% polyPs, 1.25% *Verbascum thapsus* L. and 1.25% *Thymus vulgaris* L.). Vehicle-treated cells (*i.e.,* 0.8% NaCl) were used as negative control of the treatment. The values upon the blot represent densitometry analysis of the indicated band intensities on blots from two independent experiments (Additional file 10 and Additional file 1: Figure S6 for the ‘uncutted’ gel blots). N = 2 independent experiments

For the combinatorial optimization of the nutraceutical formula, we have firstly tested the cytotoxicity of the selected compounds alone in HEK-293 T cells treated with escalating concentrations of propolis, *Thymus vulgaris* L. or *Verbascum thapsus* L. extracts for 24 h through MTS assay. Vehicle-treated cells (*i.e.,* 0.8% NaCl) were used as negative control for the experiments. The data for the single compounds are shown in Figs. 1A-D and in Additional File 1: Table S1 (*i.e.,* polyPs: 1.0405%, R^2^ = 0.9195; propolis: IC_50_ = 12.9083%, R^2^ = 0.8977; *Thymus vulgaris* L.: IC_50_ = 42.5607%, R^2^ = 0.8571; *Verbascum thapsus L.*: IC_50_ = 38.3841, R^2^ = 0.9153).

Then, we selected nontoxic doses of the single products (*i.e.,* 0.125% polyPs, 0.625% propolis, 1.25%, *Thymus vulgaris* L. and 1.25% *Verbascum thapsus* L.) to generate a formula at 1 × concentration. Thus, we tested the absence of cytotoxicity of this nutraceutical formula (Solution-3) in vitro. To this purpose, we performed MTS assay in HEK-293 T treated with escalating doses of Solution-3 for 24 h (from 0.1 to 10x), thus showing the IC_50_ at 7.1023 × concentration (R^2^ = 0.9287), as shown in Fig. 1E. The absence of cytotoxicity was further confirmed by evaluating the caspase 3 enzymatic activity examining HEK-293 T cells treated with different concentration of Solution-3 (from 0.1 to 20x) for 24 h. As positive control, HEK-293 T cells were stimulated for the same time (*i.e.,* 24 h) with a low dose of Staurosporine (*i.e.,* 0.2 μM). These data show induction of caspase 3 activity only at the highest dose of Solution-3 here tested (*i.e.,* 20x; Fig. 1F and Additional file 1: Table S2).

Based on these data, we selected the nontoxic 1 × concentration to chemically profile the Solution-3 via ultrahigh performance liquid chromatography (UHPLC) coupled with to high-resolution mass spectrometry (HRMS) using the quadrupole orbital ion trap analyzer (Q-Orbitrap; *i.e.,* UHPLC-Q-Orbitrap HRMS [60]). These analyses revealed a high total polyphenol content (TPC, *i.e.,* 1125.466 μg/g ± SD 0.514) of Solution-3, as reported in Fig. 1G and Table 1. Thus these data show a higher TPC in the formula compared to those previously reported for the single components using similar chemical profiling-based approaches (UHPLC-HRMS for propolis [61], UHPLC-QTOF-HRMS for *Thymus vulgaris* L. [62], and UPLC-MS for *Verbascum thapsus* L. [43]).

**Table 1:** The polyphenolic profile of the nutraceutical formula Solution-3 was ascertained at 1 × concentration through ultra-high-performance liquid chromatography coupled to a high-resolution Orbitrap mass spectrometry (UHPLC-Q-Orbitrap HRMS)

Analytes	1 × Solution-3	
	μg/g	± SD
Phenolic acids	**340.180**	**0.480**
Benzoic acids		
Gallic acid	14.748	0.105
Protocatechiuc acid	27.245	0.02
Cinnamic acids		
Chlorogenic acid	18.329	0.005
Ferulic acid	141.502	0.557
p-coumaric	134.178	2.637
Rosmarinic acid	3.643	0.021
Quinic acid	0.534	0.014
Flavonoids		
Flavonols	**16.631**	**0.146**
Diosmin	1.935	0.118
Quercetin	0.256	0.013
Quercetin-3b-glucoside	0.737	0.011
Rutin hydrate	7.459	0.244
Isorhamnetin-3-rutinoside	6.244	0.341
Flavones	**126.232**	**0.537**
Apigenin-7-O-glucoside	91.381	0.220
Apigenin	9.197	1.008
Luteolin	1.093	0.912
Luteolin-7-O-glucoside	24.562	0.912
Flavanones	**8.856**	**0.088**
Naringin	2.906	0.023
Naringenin	2.981	0.113
Hesperidin	2.969	0.129
Isoflavone	**633.567**	**1.319**
Daidzin	633.567	1.319
Total phenolic compounds	**1125.466**	**0.514**

The list of phenolic acids (benzoic and cinnamic acids) and flavonoids (flavonols, flavones, flavanones, isoflavones) are shown as μg/g values

The bold values represent the quantitiy (µg/g) and ±SD (µg/g) of phenolic acids, flavonols, flavones, flavanones, isoflavone and total phenolic compounds

These results suggest that this formula, due to their high TPC, may have anti-inflammatory properties due to a synergistic action of the single components. Since all the constituents of Solution-3 are already known to affect NF-kB cascade (propolis [63], *Thymus vulgaris* L. [64], *Verbascum thapsus* L. [65] and polyPs [55]), we have here tested if their combination resulted in a greater inhibition of the pathway. As expected, our immunoblotting data show a major decrease of NF-kB p65 phosphorylated at Ser311 (p[Ser311]p65-NF-kB) residues in HEK-293 T cells treated with Solution-3 (0.01 × concentration) for 24 h, as compared to the single products (*i.e.,* percentage of p[Ser311]p65-NF-kB downregulation as compared to vehicle control: 0.01 × Solution-3, 70%; 0.625% propolis, 30%; 0.125% polyPs, 60%; 1.25% *Verbascum thapsus* L., 30%; 1.25% *Thymus vulgaris* L., 20%; see Fig. 1H).

Altogether these findings indicate that this formula possesses nutraceutical benefits in terms of anti-inflammatory activity because of the synergistic activity of the single compounds present in Solution-3 at nontoxic concentration, thus also suggesting its potential antimicrobial action against several pathogens responsible for systemic inflammatory infectious diseases.

### The nutraceutical formula exerts immunomodulation by altering microbial-related pathways

**Fig. 2:**
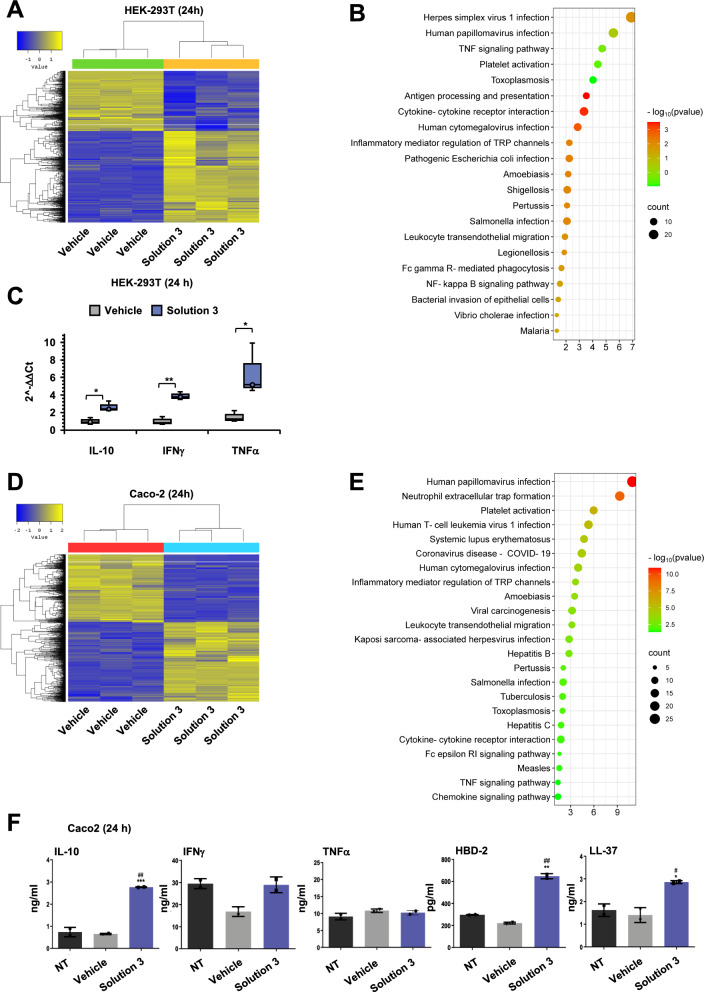
Solution-3 possesses immunomodulatory functions. **A** Heat map of the two-way Hierarchical Clustering (distance metric = Euclidean distance, linkage method = complete) using Z-score for normalized value (log2 based) representing the similarity of expression patterns between samples and genes, showing n. 1210 DEGs (Fold Change 2 and p-value < 0.05) from RNAseq analyses performed in HEK-293 T cells treated with Solution-3 (0.01x) or vehicle (0.8% NaCl) for 24 h. N = 3. See Additional file 2. **B** Bubble plot showing the results of KEGG pathway enrichment analysis obtained by using DEGs (Fold Change 2 and p-value < 0.05) from RNAseq analyses performed in HEK-293 T cells treated with Solution-3 (0.01x) or vehicle (0.8% NaCl) for 24 h. The pathways involved in microbial infection diseases and inflammation are shown. The complete list is shown in Additional file 1: Figure S1A and Additional file 3. P-values (-log10) are represented by colors, gene counts are represented by bubble size. N = 3 independent experiments per group. The chart is generated with with SRplot (http://www.bioinformatics.com.cn/plot_basic_gopathway_enrichment_bubbleplot_081_en) [137]. **(C)** Analysis of mRNA abundance normalized to b-Actin (ACTB). Data are fold-changes relative to the mRNA abundance in the control cells (2^−ΔΔCt^) for the indicated genes from real-time qPCR analysis with SYBR-Green after RNA extraction from uninfected HEK-293 T cells treated with 0.01 × Solution-3 or with 0.8% NaCl (as vehicle control) for 24 h. Data are means ± SD; *P < 0.05; **P < 0.01 by unpaired two-tailed Student’s. N = 3 independent experiments per group. See Additional file 10 for the relative expression of the genes (*i.e.,* 2^−ΔCt^ values). The graphs are generated with Microsoft Excel (version 16.82). NS, not statistic. **D** Heat map of the two-way Hierarchical Clustering (distance metric = Euclidean distance, linkage method = complete) using Z-score for normalized value (log2 based) representing the similarity of expression patterns between samples and genes, showing n. 1292 DEGs (Fold Change 2 and p-value < 0.05) from RNAseq analyses performed in Caco-2 cells treated with Solution-3 (0.01x) or vehicle (0.8% NaCl) for 24 h. N = 3. See Additional file 4. **E** Bubble plot showing the results of KEGG pathway enrichment analysis obtained by using DEGs (Fold Change 2 and p-value < 0.05) from RNAseq analyses performed in Caco-2 cells treated with Solution-3 (0.01x) or vehicle (0.8% NaCl) for 24 h. The pathways involved in microbial infection diseases, inflammation and immune-related processes are shown. The complete list is shown in Additional file 1: Figure S2A and Additional file 5. P-values (-log10) are represented by colors, gene counts are represented by bubble size. N = 3 independent experiments per group. The chart is generated with with SRplot (http://www.bioinformatics.com.cn/plot_basic_gopathway_enrichment_bubbleplot_081_en) [137].** F** Supernatants from Caco-2 cells treated with 0.01 × Solution-3 or with 0.8% NaCl (as vehicle control) for 24 h were collected and used to determine the concentration of the indicated peptides and cytokines (pg/ml or ng/ml). Untreated cells were used as negative control. Values represent the average ± SD. Data are means ± SD; *P < 0.05, **P < 0.01, ***P < 0.001 versus the vehicle-treated cells by unpaired two-tailed Student’s; ^#^P < 0.05, ^##^P < 0.01 versus the untreated cells by unpaired two-tailed Student’s. The absorbance values measured at 450 nm and the standards curves are shown in Additional file 1: Tables S4-S5. The graphs are generated with Graph Pad Prism. N = 4 independent experiments per group. NS, not statistic

To dissect the molecular pathways modulated by the nutraceutical formula, we have performed RNA sequencing (RNAseq) analysis in human cells. To this aim, HEK-293 T cells (1 × 10^6^) were treated with Solution-3 at nontoxic concentration (*i.e.,* 0.01x) for 24 h. Vehicle-treated cells (0.8% NaCl) were used as negative control for the experiments. RNAseq data showed n.1210 differentially expressed genes (DEGs; fold-change of 2, p-value < 0.05, showed in Fig. 2A and listed in Additional file 2) of which n.732 and n.478 genes were up- or down-regulated, respectively. Of interest, the Kyoto Encyclopedia of Genes and Genomes (KEGG) pathway enrichment analysis (shown in Additional file 1: Figure S1A, and listed in Additional file 3) indicated that, among those significant DEGs, there were several genes clustered in microbial-induced inflammation pathways, including infections from viruses (*e.g.,* HSV, Papilloma, Toxoplasma, Cytomegalovirus) or bacteria (*e.g., Escherichia coli, Legionella, Salmonella*), as focused on Fig. 2B, and listed in Additional file 3. Among those significantly deregulated pathways, the RNAseq data and the KEGG analyses also showed modulation of genes involved in inflammatory signaling cascades, including tumor necrosis factor (TNF), cytokine-cytokine receptors, and NF-kB signaling, thus indicating that Solution-3 may exert immunomodulatory function. Of interest, among the list of those up-regulated DEGs (Additional file 2), we found several genes taking part to the innate immune response [66], as shown in Additional file 1: Table S3.

For the above reasons, we investigated the potential modulation of inflammatory mediators involved in innate immune response exerted by Solution-3 via qPCR analyses. To this aim, we focused on some inflammatory genes mainly involved in innate immune response (*i.e.,* IL-10 [67], Interferon Gamma [IFNγ] [68], and Tumor Necrosis Factor alpha [TNFα] [69]). Moreover, the data show increased levels of IFNγ, TNFα, and IL-10 in HEK-293 T cells upon treatment with Solution-3 at 0.01 × concentration for 24 h (Fig. 2C), thus indicating a potential enhancement of innate immunity related processes in the treated cells.

To further validate this hypothesis, we performed similar experiments (*i.e*., transcriptomics RNAseq analyses) by using the human enterocyte cell line Caco-2 (intestinal epithelial cells from colon adenocarcinoma). Thus, Caco-2 cells (1 × 10^6^) were treated with Solution-3 at (0.01x) for 24 h, and vehicle-treated cells (0.8% NaCl) were used as negative control. RNAseq data showed n.1292 DEGs (fold-change of 2, p-value < 0.05, as shown in Fig. 2D and listed in Additional file 4) of which n.700 and n.592 genes were up- or down-regulated, respectively. Of interest, also in this cellular model (*i.e.,* Caco-2 cells), the Kyoto Encyclopedia of Genes and Genomes (KEGG) pathway enrichment analysis (shown in Additional file 1: Figure S2A, and listed in Additional file 5) indicated that, among those significant DEGs, there were several genes clustered in microbial-induced inflammation pathways, including infections from viruses (*e.g.,* Papilloma virus, Human T-cell leukemia virus 1, SARS-CoV-2 [*i.e.,* COVID-19], Cytomegalovirus, Herpes virus, Toxoplasma, Hepatitis B/ C viruses) or bacteria (*e.g., Salmonella, Bordetella pertussis, Mycobacterium tuberculosis*), inflammatory pathways (*i.e.,* TNF, cytokine-cytokine receptor interaction and chemokine signaling pathway) and immune-related processes (*e.g.,* neutrophil extracellular trap formation, inflammatory mediator regulation of transient receptor potential [TRP] channels and Fc epsilon RI signaling pathway), as shown in Fig. 2E. To this point, the DEGs belonging to ‘pathway in cancer’ in KEGG analyses encode for proteins that have molecular functions also related to immune response (both innate and adaptive), as shown in the protein interaction network generated using the Search Tool for the Retrieval of Interacting Genes/ Proteins (STRING) database (https://string-db.org) (see Additional file 1: Figure S2B, and listed in Additional file 6–7). In details the processes titled ‘Myeloid Leukocyte differentiation’ (GO:0002573), ‘Regulation of Leucocyte activation’ (GO:0002694) and ‘Alpha–Beta T cell differentiation’ (GO:0046632) are containing genes/ proteins involved in adaptive immunity. While, ‘Regulation of Phagocytosis’ (GO:0050764) and ‘Monuclear cell differentiation’ (GO: 1903131) molecular functions are containing genes/ proteins with activities in innate immunity (see Additional file 1: Figure S2B, and listed in Additional file 7).

Altogether, the transcriptome from RNAseq data obtained from two different cellular models (i.e., HEK-293 T [Figs. 2A, B] and Caco-2 [Figs. 2D, E] cells), further indicates immunomodulatory functions exerted by Solution-3, especially involved into innate immunity related processes.

Thus, we investigated the potential modulation of the antimicrobial peptides human beta defensin (HBD-2) and cathelicidin (LL-37) belonging to the innate immune response, together their targets IFNγ, TNFα, IL-10 [70] via ELISA assays. To this aim, we measured the amounts of the antimicrobial peptides and cytokines in the cell culture supernatant upon Solution-3 treatment at 0.01 × concentration for 24 h in Caco-2 cells via ELISA assay. The data show the ability of the nutraceutical formula to enhance the release of HBD-2, LL-37 and IL-10 in the cell culture supernatants of those Solution-3-treated Caco-2 cells, as compared to vehicle-treated or untreated cells (Fig. 2F and Additional file 1: Tables S4-S5). In contrast, the secretion of IFNγ and TNFα remained unchanged upon stimulation with Solution-3 in Caco-2 cells (Fig. 2F, Additional file 1: Tables S4-S5), as also confirmed by qPCR assays (Additional file 1: Figure S2C).

Because of the prominent role of HBD-2 and LL-37 in innate immune response against bacterial, viral and fungal invasion [71], the data here presented suggest that the treatment with the nutraceutical formula exert antimicrobial actions against several pathogens by modulating inflammatory related processes.

### The nutraceutical formula also shows bacteriostatic, bactericidal, and antifungal activities

**Fig. 3:**
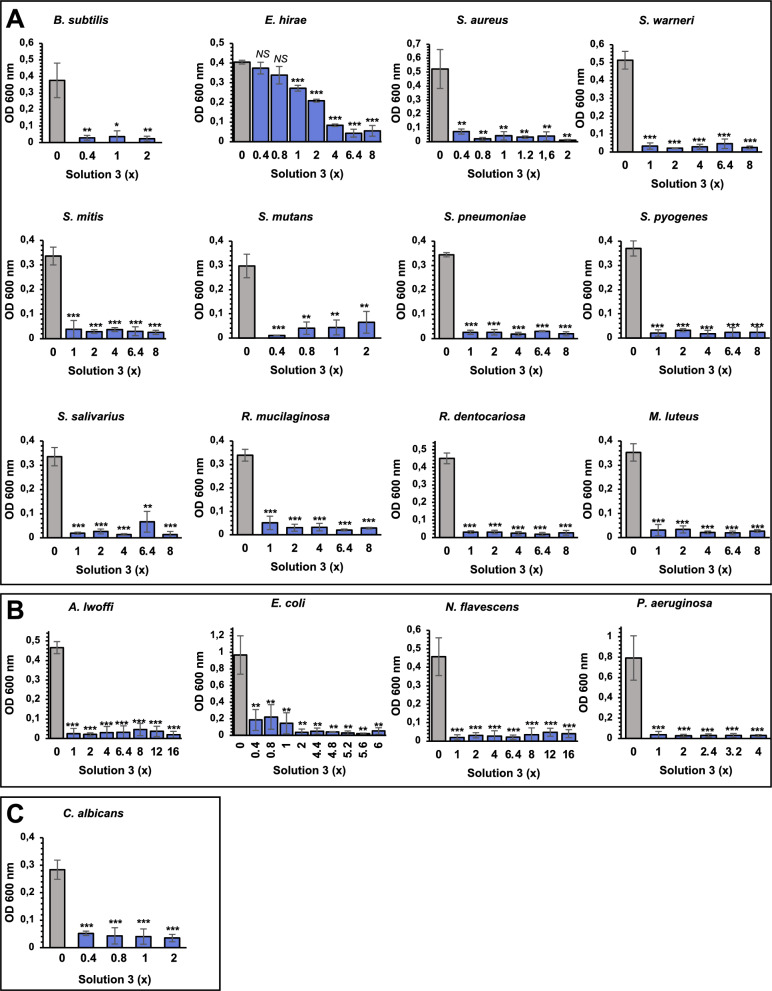
Solution-3 has bacteriostatic and bactericidal actions against bacteria (Gram positive/ negative) and fungi. **A-C** Graphs showing the bacteriostatic and bactericidal effectiveness of Solution-3 as measured by the dilution susceptibility tests [72]. Escalating concentration of Solution-3 (0.4 × to 16x) was added in the multi-well that was then incubated at 37 °C for 24 h. After incubation, the optical density at A_600 nm_ was determined. The test was performed against bacteria belonging to Gram positive (in **A**, *i.e., Staphylococcus aureus, Enterococcus hirae, Streptococcus mutans, Bacillus subtilis, Staphylococcus warneri, Streptococcus mitis, Streptococcus pneumoniae, Streptococcus pyogenes, Streptococcus salivarius, Rothia mucilaginosa, Rothia dentocariosa* and *Micrococcus luteus*), Gram negative (in **B***, i.e., Escherichia coli, Pseudomonas aeruginosa, Acinetobacter lwoffii* and *Neisseria flavescens*) and fungi (in **C**, *i.e., Candida albicans*). Data are means ± standard deviation (SD); *P < 0.05; **P < 0.01; ***P < 0.001 by unpaired two-tailed Student’s. N = 3—5 independent experiments per group. The absorbance values, as measured at 600 nm, are shown in Additional file 10. The minimum inhibitory concentrations (MICs) and the minimum bactericidal concentrations (MBCs) of Solution-3 are reported in Table 2. The graphs were generated with Microsoft Excel (version 16.82). NS, not statistic

Since RNAseq, qPCR and ELISA data indicated a modulation of some genes involved in inflammatory cascade related to microbial infection, we have tested the potential antimicrobial activity of Solution-3 against a variety of pathogens belonging to Gram positive- or negative- bacteria and fungi. We also included pathogens responsible for oral cavity and upper respiratory illnesses, and those found to co-infect with SARS-CoV-2. Thus, we used the dilution susceptibility tests (as previously described [72]) to test the effectiveness of escalating concentration of Solution-3 (from 0.4 to 16x) against pathogens belonging to Gram-positive (*Staphylococcus aureus, Enterococcus hirae, Streptococcus mutans, Bacillus subtilis, Staphylococcus warneri, Streptococcus mitis, Streptococcus pneumoniae, Streptococcus pyogenes, Streptococcus salivarius, Rothia mucilaginosa, Rothia dentocariosa and Micrococcus luteus;* see Fig. 3A and Table 2), Gram-negative bacteria (*Escherichia coli, Pseudomonas aeruginosa, Acinetobacter lwoffii and Neisseria flavescens,* see Fig. 3B and Table 2) and fungi (*Candida albicans,* see Fig. 3C and Table 2). Briefly, different doses of the nutraceutical formulation (from 0.4 to 16x) were incubated at 37 °C for 24 h to determine the optical density at A_600_ nm; subsequently the samples were spread into ‘brain heart infusion’ (BHI) or ‘trypticase soy’ (TS) agar media and incubated for 24/ 48 h for the evaluation of viable counts. Thus, the minimum inhibitory concentration (MIC) value was assigned to the lowest concentration of Solution-3, which prevents bacterial growth; while the minimum bactericidal concentration (MBC) was defined as the minimum extract concentration killing 99% of bacteria in the initial inoculum. The data show bacteriostatic activity of Solution-3 against all bacteria tested with MIC values ranging from 0.4 to 2x (except for *Enterococcus hirae*; *i.e.,* MIC = 4x; as reported in Figs. 3A-B, and Table 2). Regarding the bactericidal activities, the observed MBC values range from 0.4 to 4 × concentration (except for *Staphylococcus warneri* and *Escherichia coli*; *i.e.,* MBC: 4–8 and 6.4, respectively), as shown in Figs. 3A, B, and Table 2.

**Table 2:** The bacteriostatic and bactericidal effectiveness of escalating concentration (0.4 × to 16x) of Solution-3 was tested against bacteria belonging to Gram positive (*Staphylococcus aureus, Enterococcus hirae, Streptococcus mutans, Bacillus subtilis, Staphylococcus warneri, Streptococcus mitis, Streptococcus pneumoniae, Streptococcus pyogenes, Streptococcus salivarius, Rothia mucilaginosa, Rothia dentocariosa* and *Micrococcus luteus*), Gram negative (*Escherichia coli, Pseudomonas aeruginosa, Acinetobacter lwoffii* and *Neisseria flavescens*) and fungi (*Candida albicans*)

Genus	Species	Gram	Origin	Solution 3	
				MIC	MBC
*Bacillus*	*subtilis*	Gram +	clinical strain	0,40	0,40
*Enterococcus*	*hirae*	Gram +	DSM 3320	4,00	4,00
*Staphylococcus*	*aureus*	Gram +	ATCC 6538	0,40	0,4–1,2
*Staphylococcus*	*warneri*	Gram +	clinical strain	1,00	4–8
*Streptococcus*	*mitis*	Gram +	clinical strain	1,00	1,00
*Streptococcus*	*mutans*	Gram +	clinical strain	0,40	0,80
*Streptococcus*	*pneumoniae*	Gram +	clinical strain	1,00	1,00
*Streptococcus*	*pyogenes*	Gram +	clinical strain	1–2	1–2
*Streptococcus*	*salivarius*	Gram +	clinical strain	1,00	1–4
*Rothia*	*mucilaginosa*	Gram +	clinical strain	1,00	1–2
*Rothia*	*dentocariosa*	Gram +	clinical strain	1,00	1,00
*Micrococcus*	*luteus*	Gram +	clinical strain	1,00	1,00
*Acinetobacter*	*lwoffii*	Gram -	clinical strain	1,00	1–2
*Escherichia*	*coli*	Gram -	ATCC 13762	0,4–2	6,40
*Neisseria*	*flavescens*	Gram -	clinical strain	1,00	1–2
*Pseudomonas*	*aeruginosa*	Gram -	ATCC 27853	1,00	4,00
*Candida*	*albicans*	–	clinical strain	0,40	0,4–0,8

The genus, the gram strain, the name strain, the origin, the MIC and MBC values are shown. N = 3 independent experiments. MIC, minimal inhibitory concentration; MBC, minimal bactericidal concentration; DSM, DSMZ- German Collection of Microorganism and Cell Cultures GmbH; ATCC, American Type Culture Collection

Furthermore, the antifungal action of Solution-3 was also reported with MIC and MBC values of 0.6 × and 0.74x, respectively, against a clinical strain of *Candida albicans* (Fig. 3C and Table 2).

Altogether, these data confirmed the antimicrobial activity of the nutraceutical formula consisting in bacteriostatic and bactericidal functions against bacteria belonging to Gram-positive and -negative strains, and *Candida albicans*.

### SARS-CoV-2 inibition by nutraceutical formula is mainly driven by polyPs addition

**Fig. 4:**
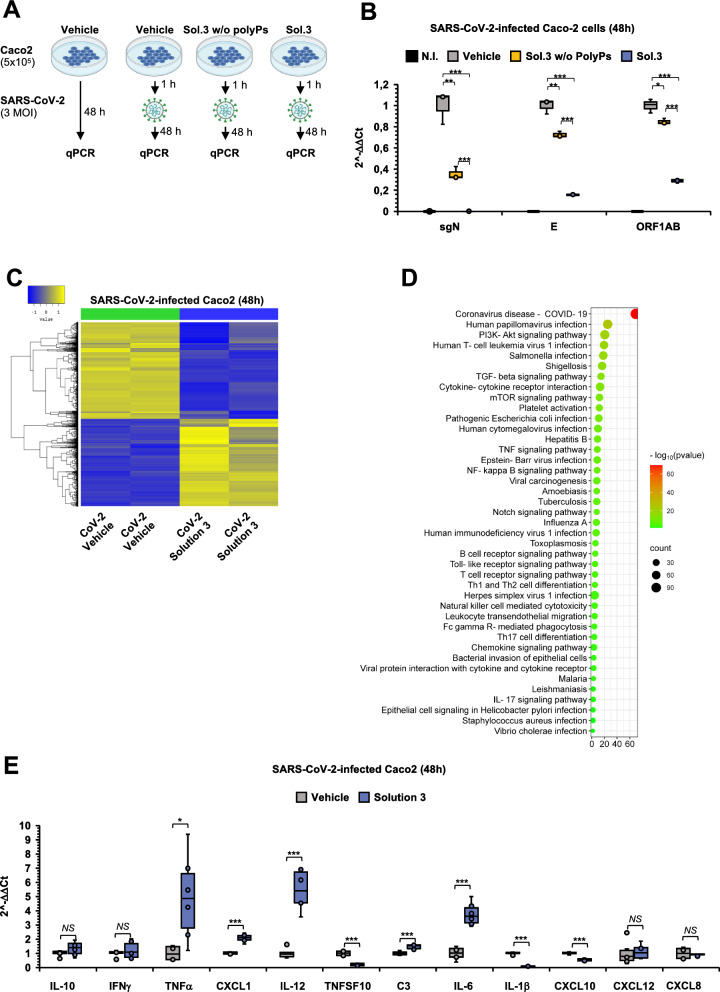
Solution-3 exerts antiviral function against SARS-CoV-2. **A** Experimental plan. Caco-2 cells were plated (5 × 10^5^ cells) and treated with Solution-3 (0.01x) or with Solution-3 without polyPs at 0.01 × concentration. After 1 h, the cells were infected with SARS-CoV-2 viral particles belonging to VOC Omicron (EG.5 sub-lineage; MOI, 3). After 48 h, the cells were lysed, and their RNA were extracted. Vehicle-treated cells (*i.e.,* 0.8% NaCl) and uninfected cells were used as negative controls for the treatment and for the infection, respectively. W/o, without. qPCR, quantitative PCR. **B** Quantification of mRNA abundance relative to that in vehicle control cells (2^−ΔΔCt^) of sgN, E and ORF1AB genes from qPCR analysis with Taqman approach. The data were normalized to human RNAse P gene. SARS-CoV-2–infected cells treated with vehicle were used as control. See Additional file 10 for the relative expression of the genes (*i.e.,* 2^−ΔCt^ values). Data are means ± SD. *p < 0.05, **p < 0.01, ***p < 0.001 by unpaired two-tailed Student’s t test; The graphs are generated with Microsoft Excel (version 16.82). N = 3 independent experiments per group. qPCR, quantitative PCR. NI, uninfected. (C) Heat map of the two-way Hierarchical Clustering (distance metric = Euclidean distance, linkage method = complete) using Z-score for normalized value (log2 based) showing n. 5353 DEGs (Fold Change 2 and raw.p-value < 0.05) from RNAseq analyses performed in SARS-CoV-2-infected Caco-2 cells pre-treated with Solution-3 (0.01x) or vehicle (0.8% NaCl). See Additional file 8 for the list of the genes. N = 2 independent experiments per group. **D** Bubble plot showing the results of KEGG pathway enrichment analysis obtained by using DEGs (Fold Change 2 and p-value < 0.05) from RNAseq analyses performed in SARS-CoV-2-tinfected-Caco-2 cells pre-treated with Solution-3 (0.01x) or vehicle (0.8% NaCl). The pathways involved in microbial infection diseases and inflammation are shown. The complete list is shown Additional file 9. The chart is generated with SRplot http://www.bioinformatics.com.cn/plot_basic_gopathway_enrichment_bubbleplot_081_en [137]. P-values (-log10) are represented by colors, gene counts are represented by bubble size. N = 2 independent experiments per group. **E** Analysis of mRNA abundance normalized to ACTB. Data are fold-changes relative to the mRNA abundance in the control cells (2^−ΔΔCt^) for the indicated genes from real-time qPCR analysis with SYBR-Green after RNA extraction from SARS-CoV-2-infected Caco-2 cells treated with 0.01 × Solution-3 or with 0.8% NaCl (as vehicle control). See Additional file 10 for the relative expression of the genes (*i.e.,* 2^−ΔCt^ values). Data are means ± SD; *P < 0.05; ***P < 0.001 by unpaired two-tailed Student’s. The graphs are generated with Microsoft Excel (version 16.82). N = 3 independent experiments per group. NS, not statistic

The nutraceutical formula here developed has been found to modulate intracellular pathways related to viral-infection diseases (Figs. 2B, E). Thus, the antiviral efficacy of Solution-3 was tested against the latest emergent XBB sub-lineages of VOC Omicron (*i.e.,* EG.5). To this purpose, we used human colorectal adenocarcinoma Caco-2 cells as cellular model of human systemic COVID-19 infection [73]. Thus, Caco-2 cells (5 × 10^4^) were treated with Solution-3 (0.01x), with Solution-3 without polyPs (0.01x) or with saline solution (0.8% NaCl) as vehicle control (for 1 h), and then infected with SARS-CoV-2 VOC Omicron (EG.5) particles at an MOI of 3 (*i.e.,* ‘prophylactic’ treatment, Fig. 4A). Uninfected cells were used as negative control for infection. After 48 h from the infection started, we measured the expression levels of viral structural Envelope (E), non-structural ORF1ab gene and sub-genomic RNA (sgN) by qPCR [74, 75]. The data show a greater inhibition of the viral genes (E, ORF1ab and sgN) in Caco-2 cells treated with Solution-3 than those treated with the same Solution-3 without polyPs (Fig. 4B). These results highlight the crucial role of polyPs in the nutraceutical formula against SARS-CoV-2. The ‘prophylactic’ antiviral effectiveness of Solution-3 were also confirmed in human HEK-293 T cells overexpressing ACE2 in plasma membrane (*i.e.,* HEK293T-ACE2 [74, 75]), as an additional cellular model of SARS-CoV-2 infection (Additional file 1: Figures S3A-B).

RNAseq analyses was then performed on the SARS-CoV-2-infected Caco-2 cells pre-treated with Solution-3 (0.01x) or with vehicle (0.8% NaCl) for 48 h (Additional file 1: Figure S3C), thus identifying n.5353 DEGs (fold-change of 2, p-value < 0.05) upon treatment with the nutraceutical formula, with n.2814 and n.2539 up- or down-regulated genes, respectively (Fig. 4C, and listed in Additional file 8). Of interest, KEGG analysis performed on those significantly DEGs identifies COVID-19 as the most statistically relevant pathway (listed in Additional file 9). Within the same analysis, we also notest modulation of genes involved in other microbial-infections (including papillomavirus, T-cell leukemia virus, *Salmonella*, *Shigella*, *Escherichia coli*, Cytomegalovirus, Hepatitis B, Epstein-Barr virus, HIV, HSV-1, Tuberculosis, Malaria, Leishmania, *Helicobacter pylori*, *Staphylococcus aureus* and *Vibro cholera*), inflammation-related signaling (including cytokine-cytokine receptors interaction, TNF, NF-kB, Toll-like receptor, chemokine signaling, T-cell receptor, IL-17, Th1-Th2 and Th17 cell differentiation), and intracellular cascades also involved in viral infections (*e.g.,* PI3K-Akt [76], TGF-β [77, 78], mTOR [76]), as shown in Fig. 4D, and listed in Additional file 9.

As far as inflammatory pathways, we investigated the expression levels of those cytokines and chemokines previously found to be modulated by Solution-3 in uninfected HEK-293 T (Fig. 2C), and Caco-2 (Fig. 2F) cells by qPCR and ELISA assays. Our data show increased of TNFα in SARS-CoV-2-infected cells pretreated with Solution-3, while in contrast the levels of IL-10 and IFNγ were unchanged (Fig. 4E). Furthermore, we also verified the expression levels of those inflammatory mediators found among the DEGs in RNAseq data obtained in SARS-CoV-2-infected cells upon Solution-3 treatment (*i.e.,* CXCL1, IL-12 and TNFSF10; Fig. 4C, D; Additional files 8–9). To this aim, our qPCR results show increased levels of CXCL1, IL-12 and reduction of TNFSF10 (Fig. 4E), thus further confirming the transcriptomic data. In additional experiments, we also investigated the potential action of the nutraceutical formula on other inflammatory genes with a role in innate immunity (*i.e.,* C3, IL-1β, IL-6, CXCL10 and CXCL12) whose modulation have been reported in COVID-19 pathogenesis and progression [79–86]. Among these genes, our qPCR data show reduced levels of IL-1β, and CXCL10, and increased expression of C3, IL-6 in those cells pre-treated with Solution-3 (Fig. 4E).

Altogether, these findings indicate that the effectiveness of the nutraceutical formula against SARS-CoV-2 is mostly due to the addition of polyPs, and that its antiviral mechanism of action also include immunomodulation.

### The nutraceutical formula exerts antiviral function against those viruses found in COVID-19 co-infections as Influenza-A and RSV-A

Since this nutraceutical formula exerts antiviral activity against SARS-CoV-2 in vitro by mostly modulating genes that are involved in the enhancement of the innate immunity, we have also investigated whether the ‘prophylactic’ action of Solution-3 could be extended to other clinically important viral pathogens.

**Fig. 5:**
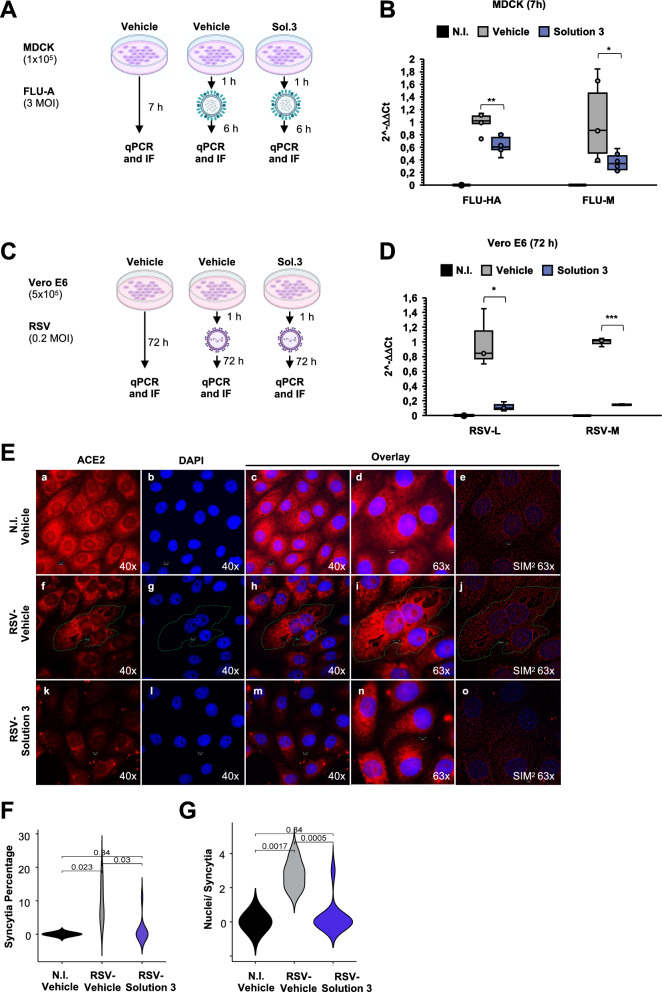
Solution-3 inhibits FLU-A and RSV-A propagation. **A** Experimental plan. MDCK cells were plated (1 × 10^5^ cells) and treated with Solution-3 (0.01x). After 1 h, the cells were infected with FLU-A viral particles (3 MOI). After 7 h, the cells were lysed for RNA extraction. Vehicle-treated cells (i.e., 0.8% NaCl) and uninfected cells were used as negative controls for the treatment and for the infection, respectively. qPCR, quantitative PCR. **B** Quantification of mRNA abundance relative to that in vehicle control cells (2^−ΔΔCt^) of viral HA and M genes from qPCR analysis with Taqman approach normalized on ACTB. FLU–A-infected cells treated with vehicle and uninfected cells were used as controls. See Additional file 10 for the relative expression of the genes (*i.e.,* 2^−ΔCt^ values). Data are means ± SD. *p < 0.05 by unpaired two-tailed Student’s t test; The graphs were generated with Microsoft Excel (version 16.82). N = 3 independent experiments per group. qPCR, quantitative PCR. NI, uninfected cells. **C** Experimental plan. Vero E6 cells were plated (5 × 10^5^ cells) and treated with Solution-3 (0.01x). After 1 h, the cells were infected with RSV viral particles (0.2 MOI). After 72 h, the cells were lysed or fixed for RNA extraction or immunofluorescence (IF) analyses, respectively. Vehicle-treated cells (*i.e.,* 0.8% NaCl) and uninfected cells were used as negative controls for the treatment and for the infection, respectively. qPCR, quantitative PCR. IF, immunofluorescence analyses. **D** Quantification of mRNA abundance relative to that in vehicle control cells (2^−ΔΔCt^) of viral L and M genes from qPCR analysis with Taqman approach normalized on RNAseP. RSV–infected cells treated with vehicle and uninfected cells were used as controls. See Additional file 10 for the relative expression of the genes (*i.e.,* 2^−ΔCt^ values). Data are means ± SD. *p < 0.05 by unpaired two-tailed Student’s t test; The graphs are generated with Microsoft Excel (version 16.82). N = 3 independent experiments per group. qPCR, quantitative PCR. NI, uninfected cells. **E** Representative immunofluorescence staining with an antibody against ACE2 (red) in Vero E6 cells treated as in **A**. DAPI was used to stain nuclei (blue). **a-e**, uninfected cells; **f**–**j**, RSV-infected cells treated with vehicle; **k**–**o**, RSV-infected cells treated with Solution-3. The images were acquired with Elyra 7 and the SIM2 images (**e**, **j**, **o**) were processed with Zeiss ZEN software (blue edition). Magnification, × 40, × 63. Scale bar, 5 μm. More than n.80 nuclei were counted (see Additional file 10). **F**–**G** Violin plots showing the percentage of syncytia (**F**) and the number of nuclei per syncytium (**G**), respectively (see Additional file 10). The quantification of the relative proportions of syncytia were performed in > 80 cells per condition. The P-value is determined by unpaired two-tailed Student’s t-test. N = 3 independent experiments per group. The graphs and P-values are obtained with SRplot (https://www.bioinformatics.com.cn/plot_basic_ggviolin_plot_113_en) [137]

Thus, we also tested the efficacy of Solution-3 against the early phases of Influenza A (FLU-A) infection, another pathogen commonly found in COVID-19 coinfection [87]. To this purpose, we used Madin-Darby canine kidney (*i.e.,* MDCK) cells as cellular model for FLU-A infection [88]. In details, MDCK (1 × 10^5^) were pre-treated with Solution-3, and then infected with FLU-A at 3 MOI for 6 h. Vehicle-treated (0.8% NaCl) and uninfected cells were respectively used as controls for treatment and infection (Fig. 5A). Our qPCR data show statistically significant decrease of viral hemagglutinin (HA) and matrix (M) genes in Solution-3-treated MDCK cells upon infection with FLU-A (Fig. 5B), thus indicating inhibition of viral replication during the early phases of infection.

Furthermore, due to the predominant role of RSV in the co-infection with SARS-CoV-2 [4, 9] especially among children [10], we have here tested the potential antiviral activity of Solution-3 towards RSV in vitro. To this purpose, Vero E6 cells (5 × 10^5^) were treated with Solution-3 (0.01x) and, after 1 h, they were infected with RSV-A viruses (belonging to A2 strain, *i.e.,* RSV-A2) at 0.2 MOI for 72 h (Fig. 5C). Vehicle-treated (0.8% NaCl) and uninfected cells were respectively used as controls for treatment and infection. Our qPCR data show statistically significant decrease of viral RNA-dependent RNA polymerase (L) and matrix (M) gene in those cells pre-treated with Solution-3 and infected with RSV-A2 (Fig. 5D). Thus, these data indicate the antiviral effectiveness of Solution-3 in impairing RSV-A infection in vitro.

RSV infection usually begins with infected cells releasing new virions nearby healthy cells and then results in cell fusion mechanisms thus resulting in multinucleated cells called syncytia [89, 90]. Thus, we have here also investigated the ability of Solution-3 to inhibit RSV propagation by breaking down the syncytia formation in infected cells [91–93]. To this aim, we performed immunofluorescence (IF) analyses on Vero E6 cells (stained with an anti-ACE2 antibody), to measure the number of cells fused in syncytia upon RSV-A infection. Our data show a mild syncytial phenotype in RSV-infected Vero E6 cells after 72 h from the infection was started, with an average of 2 nuclei per syncytia (Fig. 5E, panels f-j), as compared to uninfected cells (Fig. 5E, panels a-e; see further Additional file 1: Figure S4A for controls of the immunostaining). However, the pre-treatment with Solution-3 strongly reduced the syncytia formation in RSV-infected Vero E6 cells (Fig. 5E, panels k–o), thus resulting in decreased of both syncytia percentage (Fig. 5F, Additional file 10) and in the number of nuclei per syncytium (Fig. 5G, Additional file 10). Thus, these data indicate the effectiveness of the nutraceutical formula in impairment of RSV viral propagation by also affecting syncytia formation in vitro.

Altogether, these findings indicate the prophylactic efficacy of this nutraceutical formula during the early phases of infections against those microbial pathogens (FLU and RSV viruses) that are commonly found in COVID-19 co-infections in post-pandemic era.

## Discussion

Nutraceuticals act as dietary supplements for the prevention and treatment of several diseases, including those of infectious etiology. Indeed, several nutraceuticals, including polyphenols, are emerging as potential products to ameliorate the COVID‐19 complications mainly acting via boosting the immune system [25].

Here, we have generated a new nutraceutical formula (Solution-3) by combining extracts from natural products highly enriched in polyphenols (*i.e.,* propolis, *Verbascum thapsus* L. and *Thymus vulgaris* L.) that have been singularly described with effectiveness against different pathogens. Indeed, propolis, *Verbascum thapsus* L. and *Thymus vulgaris* L. were known for their anti-inflammatory, antioxidant and immunostimulant activities [28, 40, 46] thus resulting in antiviral, antifungal and antibacterial actions against viruses (*e.g.,* coronaviruses [30, 32, 38, 43], RSV [31, 32], adenoviruses [32], rinoviruses [32], FLU [30, 39, 44] and HSV [30, 37, 44]), bacteria (*e.g., Escherichia coli* [33, 35], *Pseudomonas aeruginosa* [29, 35, 36], *Staphylococcus aureus* [29, 33, 34, 36, 45], *Streptococcus salivarius* [36], *Streptococcus mutans* [36], and *Streptococcus pyogenes* [36]) and fungi (*e.g., Candida albicans* [36]).

The antimicrobial action of this new nutraceutical formula has been improved by the addition of polyPs in Solution-3, that are widely used as dietary additives (E452i) [47]. We have recently reported the antiviral activity of long chain length polyPs in a nebulizer system against SARS-CoV-2 by affecting virus-host cell interactions (via their binding to Spike and their driven proteome-mediated degradation of ACE2 and RdRp [55, 56]) and by impairing the cytokine storm (via inhibiting NF-kB) [55]. In addition, the antibacterial property of polyPs with benefits for chronic wounds in humans has been recently reported [53]. Of relevance for our in vitro study*,* we show that the nutraceutical formula has antimicrobial effectiveness at no cytotoxic concentration (Fig. 1E, F) we think because of its high polyphenolic content, as reported by the chemical profile of Solution-3 in Fig. 1G. Furthermore, polyPs also act as Calcium (Ca^2+^) chelators [50]. Ca^2+^ signaling has been recently discovered regulating the effectiveness of SARS-CoV-2 thought ATP2B1 cell membrane Ca^2+^-pump [138]; for this reason, we think the nutraceutical formula containing polyPs whould affect viral propagation impairing the Ca^2+^ signaling.

Thus, the efficacies of Solution-3 are triggered by the synergistic action of the single ingredients: polyPs, *Verbascum thapsus* L., *Thymus vulgaris* L. and propolis. The latest, indeed, acts synergistically with the other components modulating the inflammatory signaling pathways, thus acting as an immunomodulator, and also influencing the antioxidant status of both the infected and inflammatory activated cells, as already discussed [94].

The rate of microbial co-infections from virus, bacteria and fungi have further increased in the post-pandemics era [4]. Indeed, viral co-infections have been reported between SARS-CoV-2, FLU and RSV with the latest especially found among children < 5 years [10]. Here, we have shown the antiviral efficacy of Solution-3 against SARS-CoV-2 (Fig. 4B; Additional file 1: Figure S3B), FLU-A (Fig. 5B) and RSV-A2 (Fig. 5D–G), thus indicating the potential of the nutraceutical formula to fight viral co-infections, especially in children.

Furthermore, *S. aureus* and *C. albicans* are the most frequent Gram-positive and fungal pathogens respectively found in COVID-19 co-infections [5, 6, 11]. Also, an increase of opportunistic pathogens (*e.g., Streptococcus*, *Rothia*, *E. coli* and *Shigella*) have been reported among the changes of intestinal microbiota occurring after pandemic [7]. Of importance, Solution-3 have been here reported with anti-bacteria and antifungal actions against a variety of pathogens, including *S. aureus*, *Streptococcus* (*i.e., S. mutans, S. mitis, S. pneumoniae, S. pyogenes, S. salivarius*)*, Rothia* (*i.e., R. mucilaginosa, R. dentocariosa*), *E. coli, Shigella* and *C. albicans*, showing greater efficacy against those Gram positive bacteria in terms of bacteriostatic and bactericidal functions (see Fig. 3A–C and Table 2). Thus, the use of Solution-3 could be envisioned as a potent inhibitor to reduce microbial co-infections from viruses, bacteria and fungi, especially in those patients affected by ‘community acquired pneumonia’ (CAP) with the aim to reduce their higher risk of ‘in-hospital mortality’ particularly in elderly people because of their higher mortality rate due to CAP [95]. Furthermore, because of the antibacterial action of Solution-3 against *Staphylococcus aureus* and *Pseudomonas aeruginosa,* at this time we cannot exclude its potential effectiveness against those bacteria belonging to ESKAPE pathogens (i*.e., Enterococcus faecium, Staphylococcus aureus, Klebsiella pneumoniae, Acinetobacter baumannii, Pseudomonas aeruginosa, and Enterobacter species*) that are characterized by multiple antimicrobial resistance thus causing nosocomial infection diseases and sepsis worldwide [96]. Future studies will address in details this issue.

The severity of COVID-19 is mainly driven by immune dysregulation in the host consisting in an ‘immunosuppression phase’ [14] followed by a ‘hyperinflammatory phase’ characterized by the cytokine storm and peripheral lymphopenia augmenting the risk of co-infections [15]. Thus, the disease control in those asymptomatic/ mild patients could be ascribed by the antiviral innate immune response. This latest includes components of the complement and coagulation-fibrinolysis systems, interferons (IFN), chemokines, and cellular components, including macrophages, which lowers the viral spread targeting cytokine production and inducing the adaptive immune response (generally occurring about 2–3 weeks later) after contact with the virus [18]. Thus, a failure of innate immunity may result in an abnormal acquired immune host response that causes critical COVID-19 clinical conditions. For this reason, nutraceutical with immunostimulatory regulation may result in benefits for the treatment of COVID-19 and other infectious diseases, if administered in a particular time window of action, as we expected in a prophylactic regimen of treatment.

Here, our data obtained in uninfected cells indicate that Solution-3 enhances innate immunity related processes. Defensins are antimicrobial peptides against a variety of pathogens including bacteria, viruses and fungi. They have roles in inflammatory processes acting to stimulate antigen presenting cells (APCs), including dendritic cells maturation, thus resulting in T cell stimulation and an adaptive immune response [97]. Among its antimicrobial mechanisms of action, HBD-2 was also found to promote proinflammatory mediators, including CCL2, IL-6, IL-10 and CXCL10, to fight off infections [98]. Similarly, LL-37 exerts antiviral, antibacterial and immunomodulatory activities by stimulating cytokines/ chemokine production (*e.g.,* IL-10, IL-6 and CXCL10), leukocytes chemotaxis, differentiation of innate immune cells (including macrophages and dendritic cells) [99, 100]. IL-10 has pleiotropic immunoregulatory functions aimed by preventing excessive pro-inflammatory response from both innate and adaptive immunity [101], and high levels of IL-10 are necessary for antiviral immunity for the initial resolution of the acute phase of infections [67, 102]. Indeed, increased secretion of antimicrobial peptides HBD-2 and LL-37, together with the anti-inflammatory cytokine IL-10, have been found in epithelial Caco-2 cells upon treatment with the nutraceutical formula (Fig. 2F). Thus, the use of Solution-3, by inducing IL-10 secretion (Fig. 2C, F), represents a pharmacological strategy to enhance its immunomodulatory action by counteracting the cytokine storm triggered by hyperinflammation in COVID-19 disease. Furthermore, qPCR and RNAseq data obtained from HEK-293 T cells treated with Solution-3 show increased levels of other soluble mediators with a role in innate immunity, including IFNγ and TNFα (Fig. 2C). These findings highlight the potential role of the nutraceutical formula as ‘immunostimulant’ that may act to prevent microbial infections. However, our immunoblotting data presented in Fig. 1H show decreased levels of phosphorylated p65-NF-kB in the cells treated with Solution-3, thus indicating inhibition of the inflammatory signaling cascade. Thus, we think this is due to the active presence of the polyphenolic compounds and of the polyPs). Since NF-kB has been reported with a plethora of actions during the immune response (*i.e.,* involving both innate and adaptive immune-related processes), at this time we cannot exclude a further level of regulation exerted by Solution-3 as a potential regulator of the ‘non canonical’ NF-kB pathway through RelB” in immune cells [27]. These hypotheses need future studies to be confirmed.

Of interest, functional genomic data, including qPCR and RNAseq data analyses obtained on SARS-CoV-2-infected Caco-2 cells, also have shown immunomodulation in cells pre-treated with the Solution-3 characterized by reduced levels of IL-1β, TNFSF10 and CXCL10, and increased expression of C3, IL-6, IL-12, TNFα and CXCL1. The reduction of IL-1β may by triggered by NF-kB inhibition (Fig. 1H), whose pathway activation has been reported upon SARS-CoV-2 infection [79]. Furthermore, Solution-3 decreased CXCL10 and TNFSF10 levels in SARS-CoV-2 infected cells, whose are already recognized as biomarkers both specifically associated to infections from viral origin (Fig. 4E) [80]. On the other hand, the increased levels of C3, IL-6, IL-12, TNFα and CXCL1 as a means of activation of innate-mediated response in early infection phases have been found in SARS-CoV-2-infected Caco-2 cells (Fig. 4E). More in details, CXCL1 has been previously reported to stimulate neutrophil recruitment to inflammatory site [81]. Regarding the IL-6/ 12 family of cytokines, they are primary mediators of early innate immune responses that are involved in generation of protective immunity. They also participate in both pro- and anti-inflammatory immune-related processes, with a known impact on host-microbial responses [82, 83]. Furthermore, TNFα has been reported with a role in antiviral innate immunity in limiting the viral propagation, and its levels are increased by the nutraceutical formula upon SARS-CoV-2 infection [84]. C3 is part of the complement system that has a crucial role in innate immunity to protect the host against viral infections with several antimicrobial actions including modulation of inflammation, pathogens neutralization, enhancement of immune cells chemotaxis, and promotion of the adaptive immune response [85]. Thus, altogether our data indicate that Solution-3 has immunomodulatory properties enhancing innate immunity-related signaling processes, thus exerting potential protection against various infectious diseases caused by both emerging (*i.e.,* Zika [ZIKV], Nipah [NiV], Mpox viruses) and re-emerging viruses (Ebola [EBOV], Measles [MV, or ‘rubeola’], Dengue [DENV] and Chikungunya [CHIKV] viruses) [103].

The COVID-19 pandemic has changed the pattern of community co-circulating respiratory viruses with a resurgence of other respiratory pathogens, including RSV and FLU, resulting from a genetic bottlenecking during the COVID-19 pandemic that has led to a rise in viral genetic diversity [104]. Further, COVID-19 pandemic has impacted the landscape of childhood infectious diseases due to the ‘immunity debt’ developed by children because of the limited exposure to others infectious pathogens than SARS-CoV-2. Indeed, children, more than adults, seem to be affected by a dysregulation of the immune system, appearing weeks after the primary infection with SARS-CoV-2, thus developing the ‘Multisystem Inflammatory Syndrome in children’ (MIS-C) [105]. Furthermore, this paucity of protective immune system in children after pandemic has increased their susceptibility to other infectious diseases, often resulting in ‘tripledemic’ disease characterized by COVID-19, influenza and RSV infection [106].

Of importance, the nutraceutical formula here developed affects the viral propagation of SARS-CoV-2, FLU-A and RSV-A2 (Figs. 4B, 5B, and 5D, respectively) in prophylactic in vitro treatment. These findings suggest the potential of Solution-3 with immunomodulatory and immunostimulatory properties to be tested in children to positively modulate the immune system and to prevent or treated the early phases of the most common viral infectious diseases, including MIS-C. Furthermore, our data showing the antibacterial and antifungal properties of Solution-3, as presented in Table 2, open the way for the treatment of other childhood microbial disease, including those caused by viruses (*e.g.,* ‘herpetic stomatitis’ by HSV-1, ‘hand, foot and mouth disease’ by Enteroviruses), bacteria, fungi (*e.g.,* ‘oral thrush’ by *C. albicans*), and chronic illnesses such as asthma and rhinitis, including those of allergic origin.

Apart from respiratory infections, the COVID-19 pandemic has also caused an increase in some dermatological conditions, including psoriasis and atopic dermatitis [107], with *C. albicans* is the most common coinfect fungus in COVID-19 patients [11]. Because of the bactericidal action against *C. albicans* (Table 2), the use of Solution-3 in other nutraceutical formulations for topic applications (*e.g.,* cream) could be also helpful in treating a variety of skin infections caused by microorganisms, including bacteria, virus, fungus, or parasites [108]. Furthermore, chronic inflammatory status of the skin, by promoting oncogenic gene changes, may trigger cancer, including squamous cell carcinoma, basal cell carcinoma (*i.e.,* ‘basalioma’) and melanoma [109]. To date, topical 5-fluorouracil 5% cream [110], excision, cryosurgery, or laser surgery are used to treat basal cell carcinoma and squamous cell carcinoma of the skin [111], while radiation therapy and immunotherapy are currently used for melanoma treatment [112]. This latest, is often characterized by activation of Sonic Hedgehog (SHH) signalling, with overexpression of Gli1 and Gli2 that are responsible for the generation of an immunosuppressive tumour microenvironment [113]. Thus, the SHH inhibitors show effectiveness in Melanoma treatment [114]. Of interest, through our RNAseq data analyses obtained from uninfected HEK-293 T cells show decreased levels of Gli2 by Solution-3 (Fold Change, -2.045; p-value, 0.00004; Additional file 2). Thus, because of antimicrobial action, immunomodulation, and downregulation of SHH effectors, the use of Solution-3 may be envisioned also for the treatment of skin cancer disease. Furthermore, several head and neck tumour patients often suffer from oral morbidities followed by radiation therapy, such as oral candidiasis and oral mucositis [115]. Thus, the use of the nutraceutical formula in those patients received radiation therapy, could ameliorate the oral morbidities.

In addition, our RNAseq data analyses obtained from uninfected Caco-2 colorectal adenocarcinoma cells also show modulation of several genes belonging to the KEGG term ‘pathway in cancer’ encoding proteins that have a role in both innate and adaptive immune response (as shown in Additional file 1: Figures S2B, Additional file 7). Indeed, our data show modulation of genes/proteins involved in positive regulation of leukocyte differentiation, including α-β T cell differentiation that has been previously reported with a crucial role for the adaptive immunity [116]. Thus, the potential procedure of involving Solution-3 as adjuvant therapy for tumor management could be envisioned in the future.

Moreover, the data obtained from RNAseq analyses performed on SARS-CoV-2-infected Caco-2 cells (*i.e.,* derived from a colon carcinoma) treated with Solution-3 also show modulation of signaling cascades responsible for immunosuppressive mechanisms within the tumor microenvironment (*e.g.,* PI3K-Akt and TGF-β, as shown in Fig. 4D, and listed in Additional file 9). Thus, the use of this nutraceutical in a pill formula for systemic applications could be further envisioned as immunomodulant agent for the adjuvant therapy (during or after chemotherapeutics) with the aim to restore the innate immune response in those solid tumors characterized by immunosuppression driven by PI3K-Akt and TGF-β pathways dysregulation (*e.g.,* brain tumors, medulloblastoma [117, 118] and triple negative breast cancer [119]), once tested its pharmacokinetics and tissue biodistribution in vivo. Future studies will address this issue.

Other applications that could be taken into account for the use of the nutraceutical formula in oral infectious disease are represented by dental caries because of the antibacterial action of Solution-3 against *Streptococcus mutans*, *Rothia denterocariosa, Rothia mucilaginosa and Streptococcus salivarius* belonging to the etiopathological bacterial community involved in the cariogenic process, occurring through early childhood to old age [120, 121]. These results encourage the use of the nutraceutical formula alone or as adjuvant therapeutics in association with the currently used antibiotics [122] to decrease their doses and enhance their antimicrobial actions for the prevention and treatment of oral infectious diseases such as dental caries. Thus, this nutraceutical formula could be further proposed as a ‘proactive toothpaste’ formulation.

Here, we have shown the effectiveness of this nutraceutical against several classes of microorganisms by performing in vitro studies mainly focused on the immunomodulation. However, at this time we cannot exclude other mechanisms of action exerted by Solution-3 that are responsible for its antimicrobial activities, including antioxidant functions related to its relative amounts of polyphenols (Fig. 1G). In this regard, the viral propagation has been reported to be promoted by oxidative stress and several viruses (*e.g.,* SARS-CoV-2 [123]), once entered into the host cells, can trigger reactive oxygen species (ROS) and, subsequently, COX-2 activation [124]. Indeed, COX-2 levels have been reported as increased in response to RSV [125], FLU-A [126] and SARS-CoV-2 [127]. Here, RNAseq data analyses obtained from Caco-2-infected cells show diminished levels of COX-2 and COX-3 in those Solution-3-pre-treated cells (COX2: Fold Changes − 2.8; p-value, 6.35E-11; COX3: Fold Changes − 4.06; p-value, 3.34E-16; Additional file 8). Thus, this nutraceutical formula could be helpful in the clinical management of COVID-19 co-infections acting in substitution of COX-2-selective NSAIDs, especially for those patients that should avoid their usage (*e.g.,* children < 12 years, pregnant woman and people older than 65 years [24]) due to their adverse effects (*e.g.,* gastrointestinal bleeding, cardiovascular toxicity and nephrotoxicity). Future studies will be aimed to compare the efficacy between NSAIDs and Solution-3 in preventing or treating microbial infections.

Beyond its antiviral activity, other mechanisms of action could be also here hypothesized. In this regard, in those SARS-CoV-2 infected Caco-2 cells, we saw an increased level of Ribosomal proteins (RP) subunit proteins among the DEGs belonging to ‘COVID-19 disease’ KEGG term (Additional file 9). This is of great interest because of the underestimated role of ribosomes whose functionality is affected by the interaction with the viral non-structural proteins (NSPs, including NSP1) in order to hijack host mRNA translation thus inhibiting protein synthesis in favour of viral gene expression [128, 129]. Thus, RPs and SARS-CoV-2 interaction represent a novel target for the development of antiviral compounds. The potential of Solution-3 to affect viral spread by restoring the intracellular translation machinery will be issue of future studies with focus on ribosomal DNA action in the genome in order to better understand the interplay between infectious disease and translational machinery [130].

**Fig. 6:**
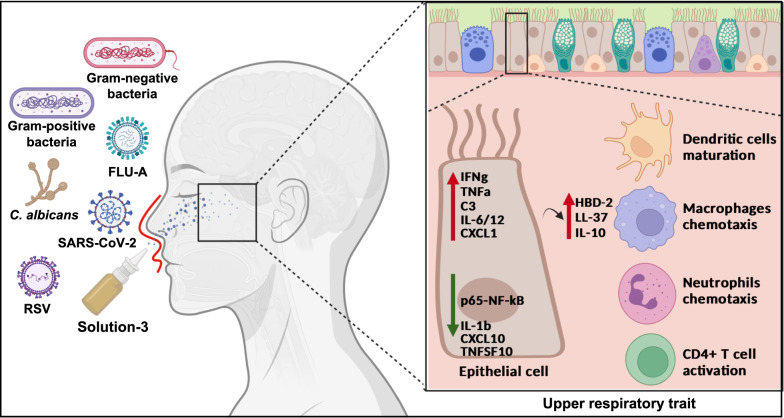
Model of action. The Solution-3 affects early phases of microbial infections by enhancing immune response. Cartoon representation to illustrate our hypothesis for the antimicrobial actions of solution 3 against SARS-CoV-2, RSV, FLU, Gram-positive and negative bacteria and *Candida albicans*. The prophylactic treatment with the nutraceutical formula results in immumomodulation in epithelial cells, including those belonging to upper respiratory traits. Indeed, Solution-3 negatively modulates NF-kB signaling cascade by reducing the phosphorylation of the NF-kB protein p65, increases the expression of inflammatory mediators IFNγ, TNFα, IL-6/ 12, CXCL1 and C3, affects the expression of IL-1β, CXCL10 and TNFSF10, and promotes the secretion of IL-10, HBD-2 and LL-37. These events may result in enhancement of innate immunity processes, including dendritic cells maturation, macrophages and neutrophils chemotaxis and CD4 + T cells activation

Altogether, our data, as summarized in Fig. 6, show a broad antimicrobial actions of a new nutraceutical formula against SARS-CoV-2, RSV, FLU, Gram-positive and negative bacteria and *Candida albicans*. The prophylactic treatment with the nutraceutical formula results in immumomodulation mostly occurring through inhibition of NF-kB signaling cascade, increased expression of inflammatory mediators IFNγ, TNFα, IL-6/ 12, CXCL1 and C3, reduction of IL-1β, CXCL10 and TNFSF10, and secretion of IL-10, HBD-2 and LL-37. These mediators are mostly involved in innate immunity response that consequently triggers dendritic cells maturation, macrophages and neutrophils chemotaxis, and CD4 + T cells activation.

Thus, the use of Solution-3 could be envisioned in several formulations for oral, topic or systemic applications, as a prophylactic strategy in order to prevent the morbidity, or to treat the early infection phases, associated to a variety of infection diseases from viral, bacterial or fungal origins, especially in children, or ‘fragile’ patients and aged people suffering from immune dysregulation in the post pandemic era.

Notwithstanding, the study here presented is performed only in vitro and is missing in vivo data in human carrying infectious diseases. As this action is demanding, in the near future the nutraceutical formula needs to address the potential side effects in human, especially considering any high doses effects of polyphenols in the formula with combined toxicity. As the fight to SARS-COV-2 are not totally ended and new pandemic would be expected in the near future, the nutraceutical formula can be a useful weapon for prophylactic and therapeutic applications.

## Conclusions

We have here developed a novel nutraceutical formula named Solution-3 possessing immunomodulatory functions responsible for a broad antimicrobial action against several classes of pathogens, including those viruses (*i.e.,* SARS-CoV-2, RSV-A and FLU-A), bacteria and fungi (*i.e., Candida Albicans*) that are currently found in co-infection diseases in post-pandemic era. The relative high polyphenolic content of the natural products (*i.e.,* propolis, *Thymus vulgaris* L. and *Verbascum thapsus* L.), together with the presence of the dietary additive long chain length polyPs, result in a positive modulation of inflammatory processes that are associated with innate immunity enhancement, thus suggesting a role for Solution-3 as immunostimulant agent that could be helpful in a prophylactic regimen, or also to fight the early phases of infection or co-infections, especially for children, aged or fragile people.

Of importance, the use of this nutraceutical formula in different formulation (*e.g.,* nano-spray, cream or pills) could be envisioned with the potential of treatment of a variety of other inflammatory-related pathological conditions, including oral infectious disease (*e.g.,* dental caries or HSV), multisystem inflammatory syndrome in children (*i.e.,* MIS-C), dermatological injury (*e.g.,* skin cancer) and also as immunostimulant agent for adjuvant therapies in solid tumors following chemotherapeutic treatment.

## Methods

### Cell culture

Vero E6 (C1008; ATCC-CRL-1586), Caco-2 (ATCC, Middlesex, UK; accession number: HTB-37 L[73]), MDCK (NBL-2; ATCC: CCL-34), HEK-293 T cells and HEK-293 T stable clones overexpressing human ACE2 (*i.e.,* HEK-293 T-ACE2[75]) were grown in a humidified 37 °C incubator with 5% CO2. The cells were cultured in feeder-free conditions using Dulbecco’s modified Eagle’s medium (41966-029; Gibco) with 10% fetal bovine serum (10270-106; Gibco), 2 mM l-glutamine (25,030–024; Gibco), and 1% penicillin/streptomycin (P0781; Sigma-Aldrich), with medium changed daily. Cells were dissociated with Trypsin–EDTA solution (T4049, Sigma-Aldrich) when the culture reached ~ 80% confluency.

### Preparation and characterization of sodium polyPs

The broad chain lengths polyPs have been produced as previously described [55] via polymerizing Sodium phosphate monobasic (NaH2PO4 ≥ 99.0%, Sigma-Aldrich) at 700 °C for 1 h without a subsequent fractional precipitation.

### Preparation of Solution-3

Solution-3 at 1 × concentration was obtained as follow: 0.125% polyPs (produced as previously described [55]), 0.625% propolis (Propolis dry extract D.E. 12% Galangin Hydrodispersible, CAS number: 85665–41-4, botanical name: Propolis, code: 010449, ACEF Spa, 29017, Italy), 1.25% *Thymus vulgaris* L. leaves, 1.25% *Verbascum thapsus L.* flowers in 0.8% Sodium Cloride (pH = 6).

### In vitro treatment

Vero E6, HEK-293 T, HEK293T-ACE2, Caco-2 and MDCK cells were plated in T25 flasks or 96 multiwells. The media were changed, and the cells were treated with polyPs (0.01 to 10%), propolis (0.5 to 5%), *Thymus vulgaris* L. leaves (0.1 to 5%), *Verbascum thapsus* L. (0.01 to 10%) or Solution-3 (0.1 to 20x). After 24 h of treatment, the cells were incubated with MTS assay, or lysed for proteins or RNA extraction. Vehicle-treated cells (*i.e.,* 0.8% NaCl) were used as the negative control for all the treatments.

### MTS assay and IC_50_ evaluation

HEK-293 T cells (1 × 10^5^) were plated in a 96-well plate and, the day after, they were treated with increasing concentrations of polyPs (0.01 to 10%), propolis (0.05 to 5%), *Thymus vulgaris* L. leaves (0.1 to 5%), *Verbascum thapsus* L. (0.01 to 10%) or Solution-3 (0.1 to 10x) for 24 h. Vehicle-treated cells (*i.e.,* 0.8% NaCl) were used as negative control. The cells were treated with MTS reagent (MTS Assay Kit, ab197010, Abcam) by directly adding it to cell culture media and incubated at 37 °C for 1 h in a humidified 37 °C incubator with 5% CO2. The absorbance was measured at 490 nm using a multimode plate reader (PerkinElmer). The absorbance values and the related folds on vehicle-treated cells are shown in Additional file 1: Table S1. The IC_50_ for polyPs, propolis, *Thymus vulgaris* L., *Verbascum thapsus* L. and Solution-3 was calculated through nonlinear regression analysis {[inhibitor] versus response (three parameters)} with ‘Quest Graph™ IC50 Calculator’ (*AAT Bioquest, Inc.*, 8 Jan. 2024; https://www.aatbio.com/tools/ic50-calculator. *N* = 6 independent experiments per group.

### Caspase 3 assay

HEK-293 T cells (1.5 × 10^5^) were plated in a 6-well plate and, the day after, they were treated with increasing concentrations of Solution-3 (0.1 to 20x). Vehicle- (*i.e.,* 0.8% NaCl) and staurosporine- (0.2 μM) treated cells were used as negative and positive control, respectively. After 24 h, the cells were washed, lysed in 200 ml/well of cell lysis buffer (PathScan^®^ Sandwich ELISA Lysis Buffer (1X) #7018, Cell Signaling Technology) left on ice for 5 min, and then stored at − 80 °C for 48 h. Then, the cell lysates were diluted in 1 × assay buffer complemented with 5 μM DTT to a final concentration of 4 mg/ml. Later, 100 μg protein lysates (25 ml) were mixed to 200 ml of 1 × assay buffer complemented with 5 μM DTT and Ac-DEVD-AMC (1:40 dilution) in a black 96-well plate. The plate was then incubated at 37 °C in the dark. The relative fluorescence units (RFU) were measured on a fluorescence plate reader with excitation at 380 nm and emission at 440 nm, using a multimode plate reader (PerkinElmer).

### Protein extraction and Immunoblotting

HEK-293 T cells were lysed in 20 mM sodium phosphate, pH 7.4, 150 mM NaCl, 10% (v/v) glycerol, 1% (w/v) sodium deoxycholate, 1% (v/v) Triton X-100, supplemented with protease inhibitors (Roche). The cell lysates were cleared by centrifugation at 16,200 × *g* for 30 min at room temperature, and the supernatants were removed and assayed for protein concentrations with Protein Assay Dye Reagent (BioRad). The cell lysates (20 μg) were resolved on 10% SDS-PAGE gels. The proteins were transferred to PVDF membranes (Millipore). After 1 h in blocking solution with 5% (w/v) dry milk fat in Tris-buffered saline containing 0.02% [v/v] Tween-20, the PVDF membranes were incubated with the primary antibodies overnight at 4 °C: anti-ACE2 (1:1000; ab15348), anti-β-actin (1:10,000; A5441; Sigma), anti-NFκB p65 [C-20] (1:500, sc-372, SantaCruz) and anti-NFkB p65 phospho S311 (1:200, sc-166748, SantaCruz). The membranes were then incubated with the required secondary antibodies for 1 h at room temperature: secondary mouse or rabbit horseradish-peroxidase-conjugated antibodies (7076S or 7074S, respectively; Cell Signaling.), diluted in 5% (w/v) milk in TBS-Tween. The protein bands were visualized by chemiluminescence detection (Pierce-Thermo Fisher Scientific Inc., IL, USA). Densitometry analysis was performed with the ImageJ software. The peak areas of the bands were measured on the densitometry plots, and the relative proportions (%) were calculated. Then, the density areas of the peaks were normalized with those of the loading controls, and the ratios for the corresponding controls are presented as fold-changes.

### Immunofluorescence analyses

Uninfected or RSV-infected-Vero E6 cells (5 × 10^4^) previously treated with Solution-3 (0.01x) or vehicle (0.8% NaCl) were fixed in 4% paraformaldehyde (PFA) in phosphate-buffered saline (PBS) for 30 min, washed three times with PBS, and permeabilized for 15 min with 0.1% Triton X-100 (215,680,010; Acros Organics) diluted in PBS. The cells were then blocked with Antibody Diluent Block (ARD1001EA, Perkin Elmer) for 1 h at room temperature (RT), and incubated overnight with the primary antibody anti-ACE2 (1:200; ab15348, Abcam) at 4 °C in a humidified chamber. The cells were washed in PBS, and incubated with anti-rabbit Alexa Fluor 546 (1:200, #A10040; ThermoFisher), as the secondary antibody. DNA was stained by incubation with 4′,6-diamidino-2- phenylindole (DAPI 1:5000; no. 62254, Thermo Fisher Scientific) for 10 min at RT. The cells were washed and mounted with coverslips with 50% glycerol (G5150, Sigma-Aldrich). Microscopy images were obtained using the Elyra 7 platform (Zeiss) with the optical Lattice SIM^2^ technology (with the ZEN software, Zeiss, black edition), using the 40 × or 63 × oil immersion objective.

### Assessment of innate peptide and cytokines production via ELISA

Caco-2 cells were stimulated with vehicle (0.8% NaCl) and Solution-3 (0.01 × concentration) for 24 h. Untreated cells exposed to only medium were used as negative control. Afterward, the supernatants were harvested and used for cytokine assay. The concentrations of HBD-2, LL-37, IFNγ, TNFα and IL-10 in cell supernatant were measured using specific human ELISA assay kits (Elabscience Biotechnology Inc. Wuhan, Hubei for HBD-2, [E-EL-H0996], LL-37 [E-EL-H2438], IFNγ [E-EL-H0108] and IL-10 [E-EL-H6154], and Abcam for TNFα [#ab181421]). The minimum detection concentrations were 62.5 pg/ml for HBD-2, 15.6 ng/ml for LL-37, 15.63 ng/ml for IFNγ, 15.63 ng/ml for TNFα and 1.56 ng/ml for IL-10. The ELISAs were conducted according to the manufacturer’s recommendations.

### SARS-CoV-2 isolation

SARS-CoV-2 (VOC OMICRON, EG.5) viruses were isolated from nasopharyngeal swabs from patients sample as previously described [55]. Briefly, Vero E6 cells (1 × 10^6^) were trypsinized and resuspended in Dulbecco’s modified Eagle’s medium (41,966–029; Gibco) with 2% FBS in T25 flask to which the clinical specimen (100 μl) was added. The inoculated cultures were grown in a humidified 37 °C incubator with 5% CO2. Seven days after infection, when cytopathic effects were observed, the cell monolayers were scrapped with the back of a pipette tip, while the cell culture supernatant containing the viral particles was aliquoted and frozen at − 80 °C. Viral lysates were used for total nucleic acid extraction for confirmatory testing and sequencing. The viral titration was made by using TaqPath COVID-19 RT-PCR Kit (A48102; Applied Biosystem).

### RSV isolation

Human respiratory syncytial virus A2 (RSV-A2, ATCC VR-1540) was isolated from Vero E6 cells. Briefly, Vero E6 cells (1 × 10^6^) were resuspended in Dulbecco’s modified Eagle’s medium (41,966–029; Gibco) with 2% FBS in T25 flask to which RSV-A2 was added. The inoculated cultures were grown in a humidified 37 °C incubator with 5% CO2. Five days after infection, the cell culture supernatant was aliquoted and frozen at − 80 °C. The viral titration was made by Reed-Muench method [131, 132]; TCDI50/ml: 2.00 × 10^3^.

### FLU-A isolation

Influenza A (FLU-A, H1N1 STRAIN A/PR/8/34, ATCC: VR-95) was isolated from MDCK cells. Briefly, MDCK cells (5 × 10^5^) were resuspended in Dulbecco’s modified Eagle’s medium (41,966–029; Gibco) with 2% FBS in T25 flask to which FLU-A was added. The inoculated cultures were grown in a humidified 37 °C incubator with 5% CO2. After 3 h, the media culture supernatant was changed and replaced with Dulbecco’s modified Eagle’s medium (41,966–029; Gibco) with 2% FBS and Trypsin, TPCK Treated (1:25 dilution; #20,233, Thermo Scientific). Five days after infection, the cell culture supernatant was aliquoted and frozen at − 80 °C. The viral titration was made by Reed-Muench method [131, 132]; TCDI50/ml: 1.51 × 10^4^.

### SARS-CoV-2 infection

Caco-2 and HEK-293 T-ACE2 cells (5 × 10^5^) were treated with 0.01 × Solution-3 or with 0.01 × Solution-3 without polyPs. Vehicle treated cells (0.8% NaCl) were used as negative control of the treatments. After 1 h, the cells were infected with SARS-CoV-2 viral particles belonging to VOC Omicron EG.5 (3 MOI) for further 48 h. Uninfected cells were used as the negative infection control. The cells were lysed, and their proteins or RNA were extracted for immunoblotting and qPCR analyses. These experiments were performed in a BLS3 authorised laboratory.

### RSV-A infection

Vero E6 cells (5 × 10^5^ for qPCR or 5 × 10^4^ for IF analyses) were plated and treated with 0.01 × Solution-3. Vehicle treated cells (0.8% NaCl) were used as negative control of the treatments. After 1 h, the cells were infected with RSV-A2 (ATCC: VR-1540) for further 72 h. Uninfected cells were used as the negative infection control. The cells were lysed of fixed for RNA extraction or Immunofluorescence (IF) analyses. These experiments were performed in a BLS3 authorised laboratory.

### FLU-A infection

MDCK cells (1 × 10^5^) were plated and treated with 0.01 × Solution-3. Vehicle treated cells (0.8% NaCl) were used as negative control of the treatments. After 1 h, the cells were infected with FLU-A (H1N1 STRAIN A/PR/8/34, ATCC: VR-95) for further 6 h. Uninfected cells were used as the negative infection control. The cells were lysed and their RNA were extracted for qPCR analyses. These experiments were performed in a BLS3 authorised laboratory.

### UHPLC-Q-orbitrap HRMS analysis

The quali-quantitative profiles of 1 × Solution-3 were performed by Ultra High-Pressure Liquid Chromatograph (UHPLC, Dionex UltiMate 3000, Thermo Fisher Scientific, Waltham, MA, USA) equipped with a degassing system, a Quaternary UHPLC pump working at 1250 bar, and an autosampler device, as previously described ([60]). Chromatographic separation was carried out with a thermostat (T = 25 ◦C) Kinetex 1.7 μm F5 (50 × 2.1 mm, Phenomenex, Torrance, CA, USA) column. The mobile phase consisted of 0.1% formic acid (FA, purchased from Merck, Darmstadt, Germany) in water (A, deionized water [< 18 MΩ x cm resistivity] obtained from a Milli-Q water purification system [Millipore, Bedford, MA, USA] and 0.1% FA in methanol (B, purchased from Merck, Darmstadt, Germany). The injection volume was 1 μL. The gradient elution program was as follows: an initial 0% B, increased to 40% B in 1 min, to 80% B in 1 min, and to 100% B in 3 min. The gradient was held for 4 min at 100% B, reduced to 0% B in 2 min, and another 2 min for column re-equilibration at 0%. The total run time was 13 min, and the flow rate was 0.5 mL/min. The mass spectrometer was operated in both negative and positive ion mode by setting 2 scan events: full ion MS and all ion fragmentation (AIF). The following settings were used in full MS mode: resolution power of 70,000 Full Width at Half Maximum (FWHM) (defined for m/z 200), scan range 80–1200 m/z, automatic gain control (AGC) target 1 × 106, injection time set to 200 ms and scan rate set at 2 scan/s. The ion source parameters were as follows: spray voltage 3.5 kV; capillary temperature 320 ◦C; S-lens RF level 60, sheath gas pressure 18, auxiliary gas 3, and auxiliary gas heater temperature 350 ◦C. For the scan event of AIF, the parameters in the positive and negative mode were set as follows: mass resolving power = 17,500 FWHM; maximum injection time = 200 ms; scan time = 0.10 s; ACG target = 1 × 105; scan range = 80–120 m/z; isolation window to 5.0 m/z; and retention time to 30 s. The collision energy was varied in the range of 10 to 60 eV to obtain representative product ion spectra. For accurate mass measurement, identification and confirmation were performed at a mass tolerance of 5 ppm for the molecular ion and for both fragments. Data analysis and processing were performed using Xcalibur software, v. 3.1.66.10 (Xcalibur, Thermo Fisher Scientific, Waltham, MA, USA).

### Microorganisms and growth conditions

The antimicrobial activity of Solution-3 was evaluated against *Staphylococcus aureus* ATCC 6538 (American Type Culture Collection, Manassas, VA), *Pseudomonas aeruginosa* ATCC 27853, *Enterococcus hirae* DSM 3320 (Leibniz Institute DSMZ-German Collection of Microorganisms and Cell Cultures GmbH), *Escherichia coli* ATCC 13762, and against clinical strains of *Streptococcus mutans*, *Bacillus subtilis*, *Candida albicans*, *Streptococcus pyogenes, Streptococcus salivarius, Staphylococcus warneri, Streptococcus mitis, Streptococcus pneumoniae, Rothia mucilaginosa, Rothia dentocariosa, Micrococcus luteus, Neisseria flavescens* and *Acinetobacter lwoffii,* [133]. The identification of clinical isolates was performed by mass spectrometry using the Matrix Assisted Laser Desorption/Ionization (MALDI) mass spectrometer (Bruker Daltonics, MALDI Biotyper, Fremont, CA, USA), a high-throughput proteomic technique for identification of a variety of bacterial and fungal species [134, 135], and biochemical phenotyping method in an BD Phoenix Automated Microbiology System (Becton Dickinson, BD Franklin Lakes, NJ, USA), according to the manufacturer’s instruction. Bacteria were cultured aerobically in broth and agar media at 37 °C. The media used were Tryptic soy (TS) (Oxoid, S.p.a., Rodano, Milano, Italy), Brain Heart Infusion (BHI) (Oxoid, S.p.a., Rodano, Milano, Italy) and Columbia CNA with 5% Sheep Blood with Colistin and Nalidixic Acid (Oxoid, S.p.a., Rodano, Milano, Italy). Microbial strains were maintained at 4 °C on agar media. The isolates were stored frozen at − 80 °C in BHI broth supplemented with 10% glycerol (v/v) (Carlo Erba, Reagents, Milan, Italy) until use and the working cultures were activated in the respective broth at 37 °C for 15–18 h.

### In Vitro antibacterial activity assays

The susceptibility of reference and clinical strains to different concentrations of Solution-3 was determined by dilution tube method of the Clinical and Laboratory Standards Institute using 1 × 10^5^ CFU/ml as standard inoculum [72] (as described by Clinical and Laboratory Standards Institute [CLSI]; available at https://clsi.org/media/tc4b1paf/m10033_samplepages-1.pdf). The formulation was added in the multi-well achieving a ranging concentration from 0.4 to 16 × and multi-well was incubated at 37 °C for 24 h. After incubation, the optical density at A_600 nm_ was determined; subsequently an aliquot of each sample was spread into BHI or TS-agar plates and then incubated for 24/ 48 h for the evaluation of viable counts. Minimum inhibitory concentration (MIC) value was assigned to the lowest concentration of Solution-3, which prevents bacterial growth. The minimum bactericidal concentration (MBC) was defined as the minimum extract concentration that killed 99% of bacteria in the initial inoculum.

### Transcriptomic analyses (RNA sequencing, RNAseq)

#### RNA isolation and library construction and sequencing

Total RNA was isolated from uninfected HEK-293 T, uninfected Caco-2 cells and SARS-CoV-2-infected Caco-2 cells by using TRIzol RNA Isolation Reagent (#15,596,018; Ambion, Thermo Fisher Scientific) was quantified in a NanoDrop™ One/One^C^ Microvolume UV–Vis Spectrophotometer (Thermo Scientific™), checked for purity and integrity and sequenced. Libraries were prepared using the TruSeq Stranded Total RNA LT Sample Prep Kit (Gold) according to the protocols recommended by the manufacturer. Trimmed reads are mapped to reference genome with HISAT2 (https://ccb.jhu.edu/software/hisat2/index.shtml), splice-aware aligner. After the read mapping, Stringtie (https://ccb.jhu.edu/software/stringtie/) was used for transcript assembly. Expression profile was calculated for each sample and transcript/gene as read count and FPKM (Fragment per Kilobase of transcript per Million mapped reads).

### Analysis of differentially expressed genes

DEG (Differentially Expressed Genes) analysis was performed on a comparison pair (Solution-3-treated- *vs* vehicle-treated cells). The read count value of known genes obtained through -e option of the StringTie was used as the original raw data. During data preprocessing, low quality transcripts are filtered. Afterwards, Trimmed Mean of M-values (TMM) Normalization were performed. Statistical analysis is performed using Fold Change, exactTest using edgeR per comparison pair. For significant lists, hierarchical clustering analysis (Euclidean Method, Complete Linkage) is performed to group the similar samples and genes. Pathway enrichment analysis was performed using KEGG database (http://www.genome.jp/kegg/). Graphs were generated by using SRplot (http://www.bioinformatics.com.cn/).

### RNA extraction and qPCR assays

Total RNA was isolated Caco-2, MDCK, HEK-293 T and HEK-293 T-ACE2 cells by using TRIzol RNA Isolation Reagent (#15,596,018; Ambion, Thermo Fisher Scientific), quantified in a NanoDrop™ One/One^C^ Microvolume UV–Vis Spectrophotometer (Thermo Scientific™). The reverse transcription was performed with SuperScript™ VILO™ cDNA Synthesis Kit (Catalog number: 11754050, ThermoFisher scientific), according to the manufacturer instructions. The reverse transcription products (cDNA) were amplified by qRT-PCR using a PCR machine (2700; Applied Biosystems, Foster City, CA, USA). The cDNA preparation was through the cycling method by incubating the complete reaction mix as follows:

- cDNA reactions: [25 °C for 10 min and 50 °C for 10 min]
- Heat-inactivation: 85 °C for 5 min
- Hold stage: 4 °C

### Human cytokines/ chemokines detection in HEK-293 T and SARS-CoV-2-Iìinfected Caco-2 cells

The targets CXCL1, CXCL10, IFNγ, IL-10, IL-6, IL-12, TNFα, C3, TNFSF10 and ACTB in uninfected HEK-293 T and Caco-2 cells and SARS-CoV-2-infected Caco-2 cells were detected with SYBR green approach by using BlastTaq 2 × qPCR MasterMix (#4891; ABM). ACTB was used as the housekeeping gene used to normalize the quantification cycle (Cq) values of the other genes. These runs were performed on a PCR machine (QuantStudio™ 5 Real-Time PCR; Applied Biosystem) with the following thermal protocol:

- Enzyme Activation: 95 °C for 3 min
- Denaturation Step: 95 °C for 15 s
- Annealing/ extension (× 40 cycles): 60 °C for 60 s

The relative expression of the target genes was determined using the 2^−ΔCt^ method, as the fold increase compared with the controls. The data are presented as means ± SD of the 2^−ΔΔCt^ values (normalized to ACTB) of three replicates. The details of the primers used in these SYBR green assays are provided below:

- ACTB Forward: GACCCAGATCATGTTTGAGACCTT
- ACTB Reverse: CCAGAGGCGTACAGGGATAGC
- IFNγ Forward: GAGTGTGGAGACCATCAAGGAAG
- IFNγ Reverse: TGCTTTGCGTTGGACATTCAAGTC
- IL-6 Forward: GCCACTCACCTCTTCAGAAC
- IL-6 Reverse: AGCATCCATCTTTTTCAGCC
- IL-10 Forward: CCTGCCTAACATGCTTCGAGA
- IL-10 Reverse: TGTCCAGCTGATCCTTCATTTG
- IL-12 Forward: TGATGGCCCTGTGCCTTAGT
- IL-12 Reverse: GGATCCATCAGAAGCTTTGCA
- TNFα Forward: TCTCTCTAATCAGCCCTCTGG
- TNFα Reverse: GCTACATGGGCTACAGGC
- CXCL1 Forward: GCGCCCAAACCGAAGTCATA
- CXCL1 Reverse: ATGGGGGATGCAGGATTGAG
- TNSF10 Forward: AGCAACACATTGTCTTCTCC
- TNSF10 Reverse: CCCACTCCTTGATGATTCCC
- C3 Forward: ACAGTGTCCTACCAAGATTCC
- C3 Reverse: TTGTAGTATGGGTGGTCTGAG

### sgN, E and ORF1ab detection in SARS-CoV-2-infected-Caco-2 and -HEK-293 T-ACE2 cells

The targets sgN, E and ORF1ab in SARS-CoV-2-infected Caco-2 and HEK-293 T-ACE2 cells were detected with Taqman approach by using SARS-CoV-2 Viral3 kit[74, 75] (CE-IVD; #infect-004, BioMol laboratories; https://www.biomollaboratories.it/). These runs were performed using 75 ng (5 μL) RNA on a PCR machine (CFX96; BioRad; in vitro diagnostics IVD approved) under the following conditions:

- UNG incubation: 25 °C for 2 min
- Reverse transcription: 50 °C for 15 min
- Inactivation/denaturation: 95 °C for 3 min
- Denaturation and annealing (for 44 cycles): [95 °C for 3 s and 60 °C for 45 s].

The relative expression of the target genes was determined using the 2^−ΔCt^ method, as the fold increase compared with the controls. The data are presented as means ± SD of the 2^−ΔΔCt^ values (normalized to RNAseP) of three replicates. The details of the primers used in these assays are provided below:

- sgN Forward: CAACCAACTTTCGATCTCTTGTA
- sgN Reverse: TCTGCTCCCTTCTGCGTAGA
- sgN Probe: 5′-FAM-ACTTCCTCAAGGAACAACATTGCCA-BBQ1-3′
- Orf1ab Forward: CCCTGTGGGTTTTACACTTAA
- Orf1ab Reverse: ACGATTGTGCATCAGCTGA
- Orf1ab Probe:5’-ROX- CCGTCTGCGGTATGTGGAAAGGTTATGG-BBQ2-3’
- E Forward: ACAGGTACGTTAATAGTTAATAGCGT
- E Reverse: ATATTGCAGCAGTACGCACACA
- E Probe: 5’ CY5-ACACTAGCCATCCTTACTGCGCTTCG BBQ2-3’
- RNAse P Forward: ATGGCGGTGTTTGCAGATTT
- RNAse P Reverse: AGCAACAACTGAATAGCCAAGG
- RNAse P Probe: 5′-HEX-TTCTGACCTGAAGGCTCTGCGCG-BHQ1-3′

### L and M detection in RSV-infected Vero E6 cells

The targets L and M in RSV-infected Vero E6 cells were detected with Taqman approach by using Taqman Multiplex MasterMix kit (#4,461,882, Applied Biosystem). These runs were performed using a PCR machine (QuantStudio™ 5 Real-Time PCR, ThermoFisher Scientific) under the following conditions:

- Hold stage: 50 °C for 2 min
- Denaturation Step: 95 °C for 10 min
- Denaturation and Annealing (× 45 cycles): [95 °C for 15 s and 60 °C for 60 s].
- Melt curve stage: [95 °C for 15 s, 60 °C for 1 min and 95 °C for 15 s]

The relative expression of the target genes was determined using the 2^−ΔCt^ method, as the fold increase compared with the controls. The data are presented as means ± SD of the 2^−ΔΔCt^ values (normalized to RNAseP) of three replicates. The details of the primers used in these assays are provided below [136]:

- VIDRL Forward: AATACAGCCAAATCCAACCAACTTTACA
- VIDRL Reverse: GCCAAGGAAGCATGCAGTAAA
- VIDRL Probe: 5′- FAM-CTTTAGTGCACAATAGCA-BHQ1-3’
- RNAseP RPP30 Forward: ATG GCG GTG TTT GCA GAC TT
- RnaseP RPP30 Reverse: AGC AAC AAC TGA ATA GCC AAG G
- RNaseP RPP30 Probe: 5′- HEX- TTC TGA CCT GAA GGC TCT GCG CG-BHQ1-3’
- M RSV Forward: GGCAAATATGGAAACATACGTGAA
- M RSV Reverse: TCTTTTTCTAAGACATTGTATTGAACAG
- M RSV PROBE: 5’-FAM-AGCTTCACGAAGGCTCCACATACACAG-BHQ1-3’

### HA and M detection in FLU-A-infected MDCK cells

The targets HA and M in FLU-A-infected MDCK cells were detected with Taqman approach by using Taqman Multiplex MasterMix kit (#4,461,882, Applied Biosystem). These runs were performed using a PCR machine (QuantStudio™ 5 Real-Time PCR, ThermoFisher Scientific) under the following conditions:

- Hold stage: 50 °C for 2 min
- Denaturation Step: 95 °C for 10 min
- Denaturation and Annealing (× 45 cycles): [95 °C for 15 s and 60 °C for 60 s].
- Melt curve stage: [95 °C for 15 s, 60 °C for 1 min and 95 °C for 15 s]

The relative expression of the target genes was determined using the 2^−ΔCt^ method, as the fold increase compared with the controls. The data are presented as means ± SD of the 2^−ΔΔCt^ values (normalized to β-Actin [ACTB, Hs_01060665, ThermoFisher Scientific]) of three replicates. The details of the primers used in these assays are provided below (adapted from ‘WHO information for the molecular detection of influenza viruses’, February 21, http://www.who.int/influenza/gisrs_laboratory/collaborating_centres/list/en/index.html):

- H1N1 HA Forward: GACACAATAATATTTGAGGCAAATGG
- H1N1 HA Reverse: GGGAGACTGCTGTTTATAGCTCC
- H1N1 HA PROBE: 5’-FAMGCTTTCGCACTGAGTAGAGGC-BHQ1-3’
- H1N1 M Forward: CTTCTAACCGAGGTCGAAACGTA
- H1N1 M Reverse: GGTGACAGGATTGGTCTTGTCTTTA
- H1N1 M PROBE: 5’-FAM-TCAGGCCCCCTCAAAGCCGAG-BHQ1

### Protein–protein interaction network

The protein interaction network was generated via the Search Tool for the Retrieval of Interacting Genes/ Proteins (STRING) database (https://string-db.org) by using a list of proteins encoded by those n.43 DEGs taking part to ‘pathway in cancer’ in KEGG pathway enrichment analyses from RNAseq data obtained in Caco-2 cells treated with Solution-3 (0.01x) or vehicle (0.8% NaCl) for 24 h. The following settings are used: minimum required interaction score: high confidence, 0.7; maximum number of interactors shown: fist shell, none—query proteins only; second shell: no more than 5 interactors.

### Statistical analysis

Statistical significance was defined as *P* < 0.05 by unpaired two-tailed Student’s *t* tests. All of the data are given as means ± SD. In the figures, statistical significance is represented as follows: *P < 0.05, **P < 0.01, and ***P < 0.001. All of the data from the qPCR assays were analyzed using unpaired two-tailed Student’s *t* tests, by comparing Solution-3-treated cells versus vehicle control–cells. ELISA data were analyzed by applying unpaired, two-tailed Student’s *t* tests to the absorbance values by comparing Solution-3- treated cells versus vehicle control or untreated cells. The background values were subtracted from the absorbance values under each condition. Caspase-3 activities were analyzed by unpaired two-tailed Student’s *t* tests by comparing RFU values measured after 1 h of incubation. The background values were subtracted from the absorbance values under each condition. The IC_50_ for polyPs, propolis, *Thymus vulgaris* L., *Verbascum thapsus* L. and Solution-3 was calculated through nonlinear regression analysis {[inhibitor] versus response (three parameters)} with ‘Quest Graph™ IC50 Calculator’ (*AAT Bioquest, Inc.*, 8 Jan. 2024; https://www.aatbio.com/tools/ic50-calculator). Statistical analysis of RNAseq is performed using Fold Change, exactTest using edgeR per comparison pair. For significant lists, hierarchical clustering analysis (Euclidean Method, Complete Linkage) is performed to group the similar samples and genes. Pathway enrichment analysis was performed using KEGG database (http://www.genome.jp/kegg/). Graphs were generated by using Microsoft Excel (v.16.82), Graph Pad Prism and SRplot (http://www.bioinformatics.com.cn/).

### Supplementary Information

**Additional file 1** (DOCX 10933 KB)

**Additional file 2**. Related to Fig. 2A. List of differentially expressed genes (DEGs) in HEK-293T cells (1x10^6^) treated with Solution-3 at 0.01x concentration for 24 hours. Vehicle-treated cells (0.08% NaCl) were used as negative control for the experiments. RNAseq analyses showed n.1210 differentially expressed genes (DEGs; fold-change of 2, p-value <0.05). Among these, n.732 and n.478 genes were found up- or down-regulated, respectively. The gene symbol, the fold-change and the p-value are shown.

**Additional file 3**. Related to Fig. 2B and S1A. List of KEGG pathway enrichment test results showing the enrichment of each gene from the gene set obtained in RNAseq analyses performed on HEK-293T cells (1x10^6^) treated with Solution-3 at 0.01x concentration for 24 hours. Vehicle-treated cells (0.08% NaCl) were used as negative control for the experiments. The pathway terms, the gene symbol, the fold-change and the p-value (from the modified fisher’s exact test) are shown.

**Additional file 4**. Related to Fig. 2D. List of differentially expressed genes (DEGs) in Caco-2 cells (1x10^6^) treated with Solution-3 at 0.01x concentration for 24 hours. Vehicle-treated cells (0.08% NaCl) were used as negative control for the experiments. RNAseq analyses showed n. 1292 differentially expressed genes (DEGs; fold-change of 2, p-value <0.05). Among these, n. 700 and n. 592 genes were found up- or down-regulated, respectively. The gene symbol, the fold-change and the p-value are shown.

**Additional file 5**. Related to Fig. 2E and S2A. List of KEGG pathway enrichment test results showing the enrichment of each gene from the gene set obtained in RNAseq analyses performed on Caco-2 cells (1x10^6^) treated with Solution-3 at 0.01x concentration for 24 hours. Vehicle-treated cells (0.08% NaCl) were used as negative control for the experiments. The pathway terms, the gene symbol, the fold-change and the p-value (from the modified fisher’s exact test) are shown.

**Additional file 6**. Related to Figure S2B. List of the proteins encoded by those DEGs belonging to ‘pathway in cancer’ as obtained from KEGG enrichment analysis on RNAseq data performed in Caco-2 cells (1x10^6^) treated with Solution-3 at 0.01x concentration or vehicle (0.8% NaCl) for 24 hours (see Additional file 5). These proteins are used as ‘input’ for the analysis with ‘Search Tool for Retrieval of Interacting Genes/Proteins’ (STRING) database. The protein names and the description of their pathways/ functions (from STRING Resources) are shown.

**Additional file 7**. Related to Figure S2B. Biological processes related to the proteins of the interaction network generated via the Search Tool for the Retrieval of Interacting Genes/ Proteins (STRING) database (https://string-db.org) by using proteins encoded by those DEGs taking part to ‘pathway in cancer’ in KEGG pathway enrichment analyses performed on RNAseq data obtained from Caco-2 cells treated with Solution-3 (0.01x) or vehicle (0.8% NaCl) for 24 hours (see Additional file 5-6). The proteins belonging to biological processes involved in immune system and inflammation are indicated in bold: positive regulation of immune system process (GO:0002684, fdr = 0.0011), inflammatory response (GO:0006954, fdr = 0.0049), leukocyte differentiation (GO:0002521, fdr = 0.0051), regulation of T cell differentiation in thymus (GO:0033081, fdr = 0.0057), response to cytokine (GO:0034097, false discovery rate [fdr] = 0.0107), mononuclear cell differentiation (GO:1903131, fdr = 0.0110) regulation of phagocytosis (GO:0050764, fdr = 0.0112), cytokine-mediated signaling pathway (GO:0019221, fdr = 0.0197), cellular response to cytokine stimulus (GO:0071345, fdr = 0.0208), myeloid leukocyte differentiation (GO:0002573, fdr = 0.0228), leukocyte migration (GO:0050900, fdr = 0.0236), positive regulation of T cell differentiation in thymus (GO:0033089, fdr = 0.0268), regulation of leukocyte activation (GO:0002694, fdr = 0.0342), alpha-beta T cell differentiation (GO:0046632, fdr = 0.0394), T cell differentiation (GO:0030217, fdr = 0.0433) and positive regulation of phagocytosis (GO:0050766, fdr = 0.0448). The term ID, the term description, the observed gene count, the background gene count, the strength, the false discovery rate (frd) and the matching proteins in the network are shown.

**Additional file 8**. Related to Figure 4C. List of differentially expressed genes (DEGs) in SARS-CoV-2-infected Caco-2 cells (5x10^5^) pre-treated with solution-3 or with vehicle (0.8% NaCl). RNAseq analyses showed n.5353 DEGs (fold-change of 2, p-value <0.05) upon treatment with the nutraceutical formula, with n.2814 and n.2539 up- or down-regulated genes, respectively. The gene symbol, the fold-change and the p-value are shown.

**Additional file 9**. Related to Fig. 4D. List of KEGG pathway enrichment test results showing the enrichment of each gene from the gene set obtained in RNAseq analyses performed on SARS-CoV-2-infected Caco-2 cells (5x10^5^) pre-treated with solution-3 or with vehicle (0.8% NaCl). The pathway terms, the gene symbol, the fold-change and the p-value (from the modified fisher’s exact test) are shown.

**Additional file 10**. Densitometry analyses related to Fig. 1H, qPCR data with the relative expression values (*i.e.,* 2^-ΔCt^) and fold on vehicle controls (*i.e.,* 2^-ΔΔCt^) related to Figs. 2C, 4B, E, 5B, D, S2C, S3B, the number and percentage of syncytia related to Figures 5E-G, and the optical density at A_600_ nm values related to Fig. 3, are shown.

## Data Availability

Data are available in the Additional file 1–10 and from the corresponding authors upon request. RNAsequencing (RNASeq) data are available on the public database (ebi.ac.uk): Uninfected HEK-293 T cells: https://www.ebi.ac.uk/fg/annotare/edit/18514/ (acc. No. E-MTAB-13918). SARS-CoV-2-infected Caco-2 cells: https://www.ebi.ac.uk/fg/annotare/edit/18517/ (acc. No. E-MTAB-13919). Uninfected Caco-2 cells: https://www.ebi.ac.uk/fg/annotare/edit/18966/ (acc. No. E-MTAB-14178).
