# Supplementary material for: Modulation of innate immunity related genes resulting in prophylactic antimicrobial and antiviral properties
Source: J Transl Med. 2024 Jun 17;22:574. doi: 10.1186/s12967-024-05378-2 (PMC11184722; doi:10.1186/s12967-024-05378-2)
Supplement: Supplementary file 1 — Additional file 1 (DOCX 10933 KB) [file 12967_2024_5378_MOESM1_ESM.docx]

**Additional File 1**

**Modulation of innate immunity related genes resulting in prophylactic antimicrobial and antiviral properties**

Veronica Ferrucci ^1,2,3^*, Marco Miceli ^2^, Chiara Pagliuca ^1^, Orazio Bianco ^2^, Luigi Castaldo ^4^, Luana Izzo ^4^, Marica Cozzolino ^2,5^, Carla Zannella ^6^, Franca Oglio ^2,5^, Antonio Polcaro ^7^, Antonio Randazzo ^4^, Roberta Colicchio ^1^, Massimiliano Galdiero ^6,8^, Roberto Berni Canani ^2,5^, Paola Salvatore ^1,2^ and Massimo Zollo ^1,2,3,9^*

^1^ Department of Molecular Medicine and Medical Biotechnology (DMMBM), University of Naples ‘Federico II’, Via Sergio Pansini 5, 80131, Naples, Italy.

^2^ CEINGE Biotecnologie Avanzate ‘Franco Salvatore’, Via Gaetano Salvatore 486, 80145, Naples, Italy.

^3^ Elysium Cell Bio Ita, Via Gaetano Salvatore 486, 80145, Naples, Italy.

^4^ Department of Pharmacy, University of Naples ‘Federico II’, Via Domenico Montesano 49, 80131, Naples, Italy.

^5^ Dipartimento di Scienze Mediche Traslazionali, University of Naples Federico II, Via Sergio Pansini 5, 80131, Naples, Italy.

^6^ Department of Experimental Medicine, University of Campania "Luigi Vanvitelli", 80138, Naples, Italy.

^7^ Polcaro Fitopreparazioni S.r.l., Via Sant Agnello, 9 D; 80030, Roccarainola, Naples, Italy.

^8^ UOC of Virology and Microbiology, University Hospital of Campania "Luigi Vanvitelli", 80138 Naples, Italy.

^9^ DAI Medicina di Laboratorio e Trasfusionale, University of Naples Federico II, Via Sergio Pansini 5, 80131, Naples, Italy.

*** Correspondences:**

*Dr. Veronica Ferrucci

Department of Molecular Medicine and Medical Biotechnology (DMMBM),

University of Naples ‘Federico II’, Via Sergio Pansini 5, 80131, Naples, Italy.

Mail: [veronica.ferrucci@unina.it](mailto:veronica.ferrucci@unina.it)

Tel: +39-081-3737904

*Prof. Massimo Zollo

Department of Molecular Medicine and Medical Biotechnology (DMMBM),

University of Naples ‘Federico II’, Via Sergio Pansini 5, 80131, Naples, Italy.

Mail: [massimo.zollo@unina.it](mailto:massimo.zollo@unina.it)

Tel: +39-081-3737875

**Additional file 1 - Supplementary Figures**

**Figure S1**

**Supplementary Figure S1. Related to Figures 2A-B. Solution-3 modulated microbial-related inflammatory pathways in HEK-293T cells. (A)** Bubble plot showing the results of KEGG pathway enrichment analysis obtained by using DEGs (Fold Change 2 and p-value <0.05) from RNAseq analyses performed in HEK-293T cells treated with Solution-3 (0.01x) or vehicle (0.8% NaCl) for 24 hours. All the statistically significant pathways are shown. See **Additional file 3** for the list of those significant pathways and genes. The bubble chart is generated with http://www.bioinformatics.com.cn/plot_basic_gopathway_enrichment_bubbleplot_081_en. [1]. P-values (-log10) are represented by colors, gene counts are represented by bubble size. N = 3 independent experiments per group.

**Figure S2**

**Supplementary Figure S2. Related to Figures 2D-E. Solution-3 modulated inflammatory pathways and immune related processes in Caco-2 cells. (A)** Bubble plot showing the results of KEGG pathway enrichment analysis obtained by using DEGs (Fold Change 2 and p-value <0.05) from RNAseq analyses performed in Caco-2 cells treated with Solution-3 (0.01x) or vehicle (0.8% NaCl) for 24 hours. All the statistically significant pathways are shown. See **Additional file 5** for the list of those significant pathways and genes. The bubble chart was generated with http://www.bioinformatics.com.cn/plot_basic_gopathway_enrichment_bubbleplot_081_en. [1]. P-values (-log10) are represented by colors, gene counts are represented by bubble size. N = 3 independent experiments per group. **(B)** Protein interaction network generated via the Search Tool for the Retrieval of Interacting Genes/ Proteins (STRING) database (https://string-db.org) by using proteins encoded by those n.43 DEGs taking part to ‘pathway in cancer’ in KEGG pathway enrichment analyses from RNAseq data obtained in Caco-2 cells treated with Solution-3 (0.01x) or vehicle (0.8% NaCl) for 24 hours. The proteins belonging to biological processes involved in immune system and inflammation are indicated: positive regulation of immune system process (dark purple, GO:0002684, fdr = 0.0011), inflammatory response (light green, GO:0006954, fdr = 0.0049), leukocyte differentiation (light purple, GO:0002521, fdr = 0.0051), regulation of T cell differentiation in thymus (very light green, GO:0033081, fdr = 0.0057), response to cytokine (light yellow, GO:0034097, false discovery rate [fdr] = 0.0107), mononuclear cell differentiation (dark grey, GO:1903131, fdr = 0.0110) regulation of phagocytosis (very dark green, GO:0050764, fdr = 0.0112), cytokine-mediated signaling pathway (dark pink, GO:0019221, fdr = 0.0197), cellular response to cytokine stimulus (dark green, GO:0071345, fdr = 0.0208), myeloid leukocyte differentiation (cyan, GO:0002573, fdr = 0.0228), leukocyte migration (dark yellow, GO:0050900, fdr = 0.0236), positive regulation of T cell differentiation in thymus (medium green, GO:0033089, fdr = 0.0268), regulation of leukocyte activation (brown, GO:0002694, fdr = 0.0342), alpha-beta T cell differentiation (red, GO:0046632, fdr = 0.0394), T cell differentiation (light pink, GO:0030217, fdr = 0.0433) and positive regulation of phagocytosis (light grey, GO:0050766, fdr = 0.0448). The network nodes represent proteins, the edges represent protein-protein associations (both functional and physical), the line color indicates the type of interaction evidence: cyan, known interaction from curated databases; pink, known interaction experimentally determined; dark green, predicted interaction from gene neighborhood; red, predicted interaction from gene fusions; dark blue, predicted interaction from from gene co-occurrence; light green, textmining; black, co-expression; ligh blue, protein homology. Minimum required interaction score: high confidence, 0.7); Maximum number of interactors shown: fist shell, none (query proteins only); second shell: no more than 5 interactors. Number of nodes: n. 48; number of edges: n. 88; average node degree: 3.67; avg. local clustering coefficient: 0.528; PPI enrichment p-value < 1.0e-16. **(C)** Analysis of mRNA abundance normalized to ACTB. Data are fold-changes relative to the mRNA abundance in the control cells (2^-ΔΔCt^) for the indicated genes from real-time qPCR analysis with SYBR-Green after RNA extraction from uninfected Caco-2 cells treated with 0.01x Solution-3 or with 0.8% NaCl (as vehicle control) for 24 hours. Data are means ± SD; See **Additional file 10** for the relative expression of the genes (*i.e.,* 2^-ΔCt^ values). The graphs were generated with Microsoft Excel (version 16.82). N = 3 independent experiments per group. NS, not statistic by unpaired two-tailed Student’s.

**Figure S3**

**Supplementary Figure S3. Related to Figure 3. Solution-3 inhibits SARS-CoV-2 by modulating inflammatory related genes. (A)** Experimental plan. HEK-293T cells overexpressing ACE2 (*i.e.,* HEK-293T-ACE2) were plated (5x10^5^ cells) and treated with Solution-3 (0.01x). After 1 hour, the cells were infected with SARS-CoV-2 viral particles VOC Omicron (EG.5 sub-lineage; MOI, 3). After 48 hours, the cells were lysed, and their RNA were extracted. Vehicle-treated cells (i.e., 0.8% NaCl) and uninfected cells were used as negative controls for the treatment and for the infection, respectively. qPCR, quantitative PCR. **(B)** Quantification of mRNA abundance relative to that in vehicle control cells (2^−ΔΔCt^) of sgN, E and ORF1AB genes from qPCR analysis with Taqman approach. SARS-CoV-2–infected cells treated with vehicle were used as control. Data are means ± SD. *p<0.05, **p<0.01, **p<0.001 by unpaired two-tailed Student’s t test; See **Additional file 10** for the relative expression of the genes (*i.e.,* 2^-ΔCt^ values). The graphs were generated with Microsoft Excel (version 16.82). N = 3 independent experiments per group. qPCR, quantitative PCR. **(C)** Experimental plan. Caco-2 cells were plated (5x10^5^ cells) and treated with Solution-3 (0.01x). After 1 hour, the cells were infected with SARS-CoV-2 viral particles VOC Omicron (EG.5 sub-lineage; MOI, 3). After 48 hours, the cells were lysed, and their RNA were extracted. Vehicle-treated cells (i.e., 0.8% NaCl) were used as negative controls for the treatment.

**Figure S4**

**Supplementary Figure S4. Related to Figure 5E. Solution-3 inhibits RSV-A syncytia formation *in vitro*. (A)** Representative immunofluorescence analysis of Vero E6 cells without the anti-ACE2 antibody (only the secondary anti-Rabbit alexa fluor 546 was used). DAPI was used to stain nuclei (blue). The images were acquired with Elyra 7 with Zeiss ZEN software (blue edition). Magnification, 40x. Scale bar, 5 μm.

**Figure S5**

** Supplementary Figure S4. Related to Figure 1H.** Immunoblotting analysis (with antibodies against the indicated proteins) of total cell lysates from HEK-293T cells treated for 24 hours with Solution-3 (0.01x), or with its components alone (*i.e.,* 0.625% propolis, 0.125% polyPs, 1.25% *Verbascum thapsus* L. and 1.25% *Thymus vulgaris* L.). Vehicle-treated cells (*i.e.,* 0.8% NaCl) were used as negative control of the treatment. N = 2 independent experiments. *T. vulgaris L.*, *Thymus vulgaris* L.; *V. thapsus* L, *Verbascum thapsus* L.; PolyPs, polyphosphates; Vehicle, NaCl.

**Additional file 1 - Supplementary Tables**

**Supplementary Table S1. Related to Figure 1A-E.** HEK-293T cells (5 × 10^4^) were treated with increasing concentrations of polyPs (0.01 to 10% μM), propolis (0.05 to 5%), *Thymus vulgaris* L. leaves (0.1 to 5%), *Verbascum thapsus* L. (0.01 to 10%) and Solution-3 (10 to 0.1x) for 24 hours. Vehicle-treated cells (*i.e.,* 0.8% NaCl) were used as negative control. The cells were incubated with MTS compound (MTS Assay Kit, ab197010, Abcam) at 37°C for 1 hour in a humidified 37°C incubator with 5% CO2. The absorbance was then measured at 490 nm using a multimode plate reader (PerkinElmer). The absorbance values and the related folds on vehicle-treated cells are shown. C, concentration; R, response.

|  | **PolyPs** | | | | | | | | | | | |
| --- | --- | --- | --- | --- | --- | --- | --- | --- | --- | --- | --- | --- |
|  | **490 nm** | | | | | | **Fold on Vehicle** | | | | | |
| **C (%)** | **R1** | **R2** | **R3** | **R4** | **R5** | **R6** | **R1** | **R2** | **R3** | **R4** | **R5** | **R6** |
| **0** | 0,749 | 0,745 | 0,797 | 0,799 | 0,774 | 0,7728 | 0,969 | 0,964 | 1,031 | 1,034 | 1,002 | 1,000 |
| **10** | 0,149 | 0,149 | 0,151 | 0,147 | 0,152 | 0,149 | 0,193 | 0,193 | 0,195 | 0,190 | 0,197 | 0,193 |
| **5** | 0,248 | 0,242 | 0,251 | 0,25 | 0,251 | 0,256 | 0,321 | 0,313 | 0,325 | 0,323 | 0,325 | 0,331 |
| **1** | 0,395 | 0,392 | 0,391 | 0,385 | 0,384 | 0,393 | 0,511 | 0,507 | 0,506 | 0,498 | 0,497 | 0,509 |
| **0,5** | 0,402 | 0,424 | 0,41 | 0,402 | 0,407 | 0,41 | 0,520 | 0,549 | 0,531 | 0,520 | 0,527 | 0,531 |
| **0,1** | 0,741 | 0,654 | 0,711 | 0,647 | 0,677 | 0,733 | 0,959 | 0.846 | 0,920 | 0.837 | 0.876 | 0,948 |
| **0,05** | 0,704 | 0,69 | 0,702 | 0,759 | 0,681 | 0,714 | 0,911 | 0.893 | 0,908 | 0,982 | 0.881 | 0,924 |
| **0,01** | 0,675 | 0,737 | 0,708 | 0,703 | 0,726 | 0,713 | 0.873 | 0,954 | 0,916 | 0,910 | 0,939 | 0,923 |
|  | **Propolis** | | | | | | | | | | | |
|  | **490 nm** | | | | | | **Fold on Vehicle** | | | | | |
| **C (%)** | **R1** | **R2** | **R3** | **R4** | **R5** | **R6** | **R1** | **R2** | **R3** | **R4** | **R5** | **R6** |
| **0** | 0,612 | 0,608 | 0,619 | 0,600 | 0,608 | 0,627 | 0,992 | 0,985 | 1,002 | 0,972 | 0,985 | 1,016 |
| **5** | 0,355 | 0,368 | 0,365 | 0,369 | 0,364 | 0,368 | 0,576 | 0,596 | 0,592 | 0,598 | 0,590 | 0,597 |
| **2,5** | 0,450 | 0,468 | 0,452 | 0,467 | 0,464 | 0,468 | 0,729 | 0,758 | 0,733 | 0,757 | 0,752 | 0,759 |
| **1** | 0,520 | 0,509 | 0,507 | 0,476 | 0,505 | 0,517 | 0.843 | 0.825 | 0.822 | 0,772 | 0.818 | 0.837 |
| **0,5** | 0,504 | 0,520 | 0,526 | 0,527 | 0,543 | 0,519 | 0.818 | 0.843 | 0.853 | 0.855 | 0.880 | 0.841 |
| **0,25** | 0,526 | 0,504 | 0,526 | 0,535 | 0,541 | 0,551 | 0.852 | 0.817 | 0.852 | 0.867 | 0.877 | 0.892 |
| **0,1** | 0,621 | 0,556 | 0,560 | 0,549 | 0,560 | 0,565 | 1,006 | 0,901 | 0,907 | 0.890 | 0,907 | 0,916 |
| **0,05** | 0,578 | 0,581 | 0,582 | 0,582 | 0,576 | 0,589 | 0,937 | 0,941 | 0,943 | 0,943 | 0,933 | 0,954 |
|  | ***Thymus vulgaris* L.** | | | | | | | | | | | |
|  | **490 nm** | | | | | | **Fold on Vehicle** | | | | | |
| **C (%)** | **R1** | **R2** | **R3** | **R4** | **R5** | **R6** | **R1** | **R2** | **R3** | **R4** | **R5** | **R6** |
| **0** | 0,992 | 0,958 | 0,974 | 0,963 | 0,983 | 0,978 | 1,018 | 0,983 | 0,999 | 0,988 | 1,009 | 1,003 |
| **5** | 0,713 | 0,717 | 0,711 | 0,716 | 0,726 | 0,734 | 0,732 | 0,736 | 0,729 | 0,735 | 0,745 | 0,753 |
| **2,5** | 0,753 | 0,762 | 0,765 | 0,76 | 0,731 | 0,783 | 0,773 | 0,782 | 0,785 | 0,780 | 0,750 | 0.803 |
| **1** | 0.802 | 0,964 | 0,733 | 0,724 | 0,732 | 0,748 | 0.823 | 0,989 | 0,752 | 0,743 | 0,751 | 0,767 |
| **0,5** | 0,984 | 0,762 | 0.832 | 0,79 | 0,719 | 0,712 | 1,010 | 0,782 | 0.854 | 0.811 | 0,738 | 0,731 |
| **0,25** | 0.855 | 0,782 | 0.868 | 0.827 | 0.851 | 0.827 | 0.877 | 0.802 | 0.891 | 0.848 | 0.873 | 0.848 |
| **0,1** | 0.869 | 0.834 | 0.849 | 0.817 | 0.843 | 0.831 | 0.892 | 0.856 | 0.871 | 0.838 | 0.865 | 0.853 |
|  | ***Verbascum Thapsus* L.** | | | | | | | | | | | |
|  | **490 nm** | | | | | | **Fold on Vehicle** | | | | | |
| **C (%)** | **R1** | **R2** | **R3** | **R4** | **R5** | **R6** | **R1** | **R2** | **R3** | **R4** | **R5** | **R6** |
| **0** | 0,745 | 0,755 | 0.826 | 0,932 | 0.862 | 0.879 | 0.894 | 0,906 | 0,991 | 1,119 | 1,035 | 1,055 |
| **10** | 0,774 | 0,753 | 0,717 | 0,711 | 0,7 | 0,732 | 0,929 | 0,904 | 0.861 | 0.853 | 0.840 | 0.879 |
| **5** | 0,744 | 0,707 | 0,782 | 0,792 | 0,735 | 0.851 | 0.893 | 0.849 | 0,939 | 0,951 | 0.882 | 1,021 |
| **1** | 0,762 | 0.805 | 0,798 | 0,765 | 0,731 | 0,784 | 0,915 | 0,966 | 0,958 | 0,918 | 0.877 | 0,941 |
| **0,5** | 0,741 | 0.803 | 0.856 | 0.825 | 0,752 | 0,765 | 0.889 | 0,964 | 1,027 | 0,990 | 0,903 | 0,918 |
| **0,1** | 0,787 | 0.818 | 0.811 | 0,794 | 0.806 | 0.843 | 0,945 | 0,982 | 0,973 | 0,953 | 0,967 | 1,012 |
| **0,05** | 0,791 | 0.84 | 0.847 | 0.801 | 0.886 | 0.86 | 0,949 | 1,008 | 1,017 | 0,961 | 1,063 | 1,032 |
| **0,01** | 0,779 | 0.827 | 0.847 | 0.862 | 0.818 | 0.886 | 0,935 | 0,993 | 1,017 | 1,035 | 0,982 | 1,063 |
|  | **Solution-3** | | | | | | | | | | | |
|  | **490 nm** | | | | | | **Fold on Vehicle** | | | | | |
| **C (x)** | **R1** | **R2** | **R3** | **R4** | **R5** | **R6** | **R1** | **R2** | **R3** | **R4** | **R5** | **R6** |
| **0** | 0,513 | 0,587 | 0,605 | 0,565 | 0,586 | 0,564 | 0,904 | 1,034 | 1,066 | 0,995 | 1,032 | 0,992 |
| **10** | 0,288 | 0,293 | 0,291 | 0,287 | 0,291 | 0,291 | 0,507 | 0,516 | 0,513 | 0,505 | 0,513 | 0,512 |
| **5** | 0,316 | 0,327 | 0,321 | 0,326 | 0,335 | 0,322 | 0,556 | 0,576 | 0,566 | 0,574 | 0,589 | 0,567 |
| **2,5** | 0,369 | 0,390 | 0,389 | 0,386 | 0,402 | 0,391 | 0,649 | 0,687 | 0,685 | 0,679 | 0,708 | 0,689 |
| **1** | 0,476 | 0,466 | 0,434 | 0,472 | 0,447 | 0,460 | 0.838 | 0.820 | 0,764 | 0.831 | 0,788 | 0.810 |
| **0,5** | 0,521 | 0,463 | 0,467 | 0,512 | 0,481 | 0,496 | 0,918 | 0.815 | 0.822 | 0,903 | 0.847 | 0.873 |
| **0,25** | 0,562 | 0,541 | 0,553 | 0,566 | 0,574 | 0,576 | 0,990 | 0,953 | 0,974 | 0,998 | 1,012 | 1,014 |
| **0,1** | 0,607 | 0,613 | 0,612 | 0,618 | 0,597 | 0,636 | 1,069 | 1,080 | 1,079 | 1,089 | 1,052 | 1,120 |

**Supplementary Table S2. Related to Figure 1F.** HEK-293T cells (1.5 × 10^5^) were treated with increasing concentrations of Solution-3 (0.1 to 20x) for 24 hours. Vehicle-treated cells (*i.e.,* 0.8% NaCl) were used as negative control. Staurisporine-treated cells (i.e., 0.2 μM) were used as positive control of the assay. The cells were incubated with the caspase-3 fluorogenic substrate (N-Acetyl-Asp-Glu-Val-Asp-7-amino-4-methylcoumarin or Ac-DEVD-AMC) at 37ºC in the dark. The relative fluorescent units (RFUs) were acquired at 1 hour from the incubation started with a using a multimode plate reader (PerkinElmer) with excitation at 380 nm and emission at 440 nm. The values are shown as folds on vehicle-treated cells. The RFUs values and the related folds on vehicle-treated cells are shown. C, concentration; R, response. Exc, excitation; Em, Emission

|  |  | **RFU (Exc-Em 380-440)** | | | | | | | | |
| --- | --- | --- | --- | --- | --- | --- | --- | --- | --- | --- |
|  | **C** | **R1** | **R2** | **R3** | **R4** | **R5** | **R6** | **R7** | **R8** | **R9** |
| **NaCl** | **0.80%** | 21945 | 22021 | 23746 | 28698 | 28384 | 26632 | 32133 | 30383 | 31171 |
| **Staurisporine** | **0,2 μM** | 34790 | 31856 | 32587 | 33022 | 32616 | 34605 | 34378 | 32358 | 31513 |
| **Solution-3** | **0,1x** | 23847 | 23782 | 26124 | 26927 | 26518 | 26353 | 28391 | 25654 | 27763 |
|  | **1x** | 26158 | 27888 | 28265 | 28304 | 28411 | 29836 | 28627 | 27232 | 26628 |
|  | **10x** | 26564 | 26497 | 29549 | 25351 | 25599 | 24895 | 31233 | 30700 | 29860 |
|  | **20x** | 33480 | 34688 | 31146 | 28816 | 28574 | 28713 | 37689 | 37112 | 35259 |
|  |  | **Fold on Vehicle (NaCl)** | | | | | | | | |
|  | **C** | **R1** | **R2** | **R3** | **R4** | **R5** | **R6** | **R7** | **R8** | **R9** |
| **Vehicle (NaCl)** | **0.80%** | 0.806 | 0.809 | 0.872 | 1,054 | 1,042 | 0,978 | 1,180 | 1,116 | 1,145 |
| **Staurisporine** | **0,2 μM** | 1,277 | 1,170 | 1,197 | 1,212 | 1,198 | 1,271 | 1,262 | 1,188 | 1,157 |
| **Solution-3** | **0,1x** | 0.876 | 0.873 | 0,959 | 0,989 | 0,974 | 0,968 | 1,042 | 0,942 | 1,019 |
|  | **1x** | 0,960 | 1,024 | 1,038 | 1,039 | 1,043 | 1,096 | 1,051 | 1,000 | 0,978 |
|  | **10x** | 0,975 | 0,973 | 1,085 | 0,931 | 0,940 | 0,914 | 1,147 | 1,127 | 1,096 |
|  | **20x** | 1,229 | 1,274 | 1,144 | 1,058 | 1,049 | 1,054 | 1,384 | 1,363 | 1,295 |

**Supplementary Table S3. Related to Figure 2A.** List of those upregulated genes belonging to innate immune response signature [2] from RNAseq analyses performed in HEK-293T cells (1x10^6^) treated with Solution-3 at 0.01x concentration for 24 hours. Vehicle-treated cells (0.08% NaCl) were used as negative control for the experiments. The gene symbol, the description, the fold-change (FC) and the p-value are shown.

| **Gene_Symbol** | **Description** | **FC** | **p-value** |
| --- | --- | --- | --- |
| AKNA | AT-hook transcription factor | 13,154514 | 1,02151E-51 |
| DDIT3 | DNA damage inducible transcript 3 | 11,634716 | 8,3676E-120 |
| TRIB3 | tribbles pseudokinase 3 | 9,879473 | 2,07202E-96 |
| ATF3 | activating transcription factor 3 | 9,455325 | 5,40067E-34 |
| HMOX1 | heme oxygenase 1 | 8,482049 | 3,5303E-107 |
| C5 | complement C5 | 6,606813 | 1,04624E-27 |
| XBP1 | X-box binding protein 1 | 4,271442 | 6,36837E-44 |
| CEBPB | CCAAT enhancer binding protein beta | 3,553496 | 1,51577E-22 |
| INPP5D | inositol polyphosphate-5-phosphatase D | 3,495716 | 4,51598E-07 |
| VLDLR | very low density lipoprotein receptor | 3,404214 | 1,85368E-18 |
| IL23A | interleukin 23 subunit alpha | 3,166886 | 8,43258E-08 |
| SLC2A11 | solute carrier family 2 member 11 | 3,040654 | 8,99045E-14 |
| IRAK3 | interleukin 1 receptor associated kinase 3 | 2,916126 | 0,000187172 |
| C2 | complement C2 | 2,889219 | 1,61245E-08 |
| EIF4EBP1 | eukaryotic translation initiation factor 4E binding protein 1 | 2,877647 | 4,64952E-29 |
| EGF | epidermal growth factor | 2,841458 | 0,000160389 |
| ANXA1 | annexin A1 | 2,826123 | 1,00485E-11 |
| VEGFA | vascular endothelial growth factor A | 2,712263 | 1,5065E-31 |
| PTK2B | protein tyrosine kinase 2 beta | 2,562043 | 1,8874E-07 |
| MAPK10 | mitogen-activated protein kinase 10 | 2,529409 | 3,62398E-07 |
| NLRP1 | NLR family pyrin domain containing 1 | 2,512069 | 0,000584145 |
| MR1 | major histocompatibility complex, class I-related | 2,400502 | 8,4697E-12 |
| GAB2 | GRB2 associated binding protein 2 | 2,391433 | 1,007E-10 |
| IFRD1 | interferon related developmental regulator 1 | 2,334863 | 5,82968E-23 |
| ZBTB20 | zinc finger and BTB domain containing 20 | 2,331448 | 2,42684E-05 |
| CDKN1A | cyclin dependent kinase inhibitor 1A | 2,322247 | 1,37308E-17 |
| TRIM66 | tripartite motif containing 66 | 2,266373 | 4,83008E-06 |
| RELB | RELB proto-oncogene, NF-kB subunit | 2,253914 | 4,51304E-06 |
| TRAF1 | TNF receptor associated factor 1 | 2,224136 | 8,43801E-05 |
| DUSP16 | dual specificity phosphatase 16 | 2,180753 | 3,41113E-16 |
| NFIL3 | nuclear factor, interleukin 3 regulated | 2,159441 | 7,9882E-08 |
| ERN1 | endoplasmic reticulum to nucleus signaling 1 | 2,083349 | 1,01797E-07 |
| TRIM5 | tripartite motif containing 5 | 2,067453 | 5,16433E-07 |
| PTGS2 | prostaglandin-endoperoxide synthase 2 | 2,021154 | 3,33555E-05 |
| TSC22D3 | TSC22 domain family member 3 | 2,001722 | 9,29827E-08 |

**Supplementary Table S4**. **Related to Figure 2F.** Data show the absorbance at 450 nm for the samples (standards, ST) used for the standard curves related to the ELISA assays described in **Figure 2F** for the indicated peptides and cytokines (Envision plate reader; Perkin Elmer). The concentrations of the standards are shown as pg/ml or ng/ml. ST, standard.

|  | **HBD-2** | | **LL-37** | | **IFNγ** | | **TNFα** | | **IL-10** | |
| --- | --- | --- | --- | --- | --- | --- | --- | --- | --- | --- |
|  | **pg/ml** | **450 nm** | **ng/ml** | **450 nm** | **ng/ml** | **450 nm** | **ng/ml** | **450 nm** | **ng/ml** | **450 nm** |
| **ST1** | 4000 | 1,206 | 100 | 3,195 | 1000 | 3,38 | 1000 | 3,262 | 100 | 2,72 |
| **ST2** | 2000 | 0,658 | 50 | 2,613 | 500 | 3,26 | 500 | 1,9 | 50 | 1,72 |
| **ST3** | 1000 | 0,298 | 25 | 1,811 | 250 | 2,403 | 250 | 1,048 | 25 | 1,02 |
| **ST4** | 500 | 0,155 | 12,5 | 1,112 | 125 | 1,621 | 125 | 0,538 | 12,5 | 0,54 |
| **ST5** | 250 | 0,099 | 6,25 | 0,533 | 62,5 | 0,95 | 62,5 | 0,312 | 6,25 | 0,297 |
| **ST6** | 125 | 0,062 | 3,13 | 0,3 | 31,25 | 0,568 | 31,25 | 0,197 | 3,13 | 0,175 |
| **ST7** | 62,5 | 0,051 | 1,56 | 0,25 | 15,63 | 0,371 | 15,63 | 0,125 | 1,56 | 0,114 |
| **ST8** | 0 | 0 | 0 | 0 | 0 | 0 | 0 | 0 | 0 | 0 |

**Supplementary Table S5. Related to Figure 2F.** Data show the absorbance at 450 nm in the ELISA assays described in **Figure 2F** for the indicated peptides and cytokines (Envision plate reader; Perkin Elmer) evaluated in the cell culture supernatant of Caco-2 cells treated for 24 hours with Solution-3 (0.01x concentration) or vehicle (0.8% NaCl). Untreated (NT) cells were used as negative controls. The absorbance values, their average and the concentration (pg/ml or ng/ml) resulting from the interpolation with the standard curves for each peptide or cytokines (see **Supplementary Table 3**) are shown. NT, untreated cells.

|  | **HBD-2** | | | **LL-37** | | | **IL-10** | | |
| --- | --- | --- | --- | --- | --- | --- | --- | --- | --- |
|  | **450 nm** | **Average (450 nm)** | **Interpolation (*pg/ml*)** | **450 nm** | **Average (450 nm)** | **Interpolation (ng*/ml*)** | **450 nm** | **Average (450 nm)** | **Interpolation (ng*/ml*)** |
| **NT** | 0.098 | 0.098 | 295.589 | 0.18 | 0.2 | 1.821 | 0.044 | 0.048 | 0.586 |
|  | 0.098 |  |  | 0.22 |  |  | 0.052 |  |  |
|  | 1 | 0.099 | 299.343 | 0.163 | 0.163 | 1.429 | 0.054 | 0.062 | 0.890 |
|  | 0.098 |  |  | 0.163 |  |  | 0.07 |  |  |
| **Vehicle** | 0.078 | 0.08 | 226.935 | 0.18 | 0.183 | 1.640 | 0.052 | 0.052 | 0.673 |
|  | 0.082 |  |  | 0.186 |  |  | 0.052 |  |  |
|  | 0.075 | 0.077 | 215.279 | 0.137 | 0.139 | 1.176 | 0.045 | 0.05 | 0.629 |
|  | 0.079 |  |  | 0.141 |  |  | 0.055 |  |  |
| **Solution-3** | 0.193 | 0.193 | 630.050 | 0.27 | 0.3 | 2.902 | 0.148 | 0.148 | 2.784 |
|  | 0.193 |  |  | 0.33 |  |  | 0.148 |  |  |
|  | 0.201 | 0.203 | 663.178 | 0.292 | 0.292 | 2.815 | 0.148 | 0.147 | 2.761 |
|  | 0.205 |  |  | 0.292 |  |  | 0.146 |  |  |
|  | **IFNγ** | | | **TNFα** | | |  |  |  |
|  | **450 nm** | **Average (450 nm)** | **Interpolation (ng*/ml*)** | **450 nm** | **Average (450 nm)** | **Interpolation (ng*/ml*)** |  |  |  |
| **NT** | 0.526 | 0.528 | 31.054 | 0.07 | 0.079 | 9.789 |  |  |  |
|  | 0.53 |  |  | 0.088 |  |  |  |  |  |
|  | 0.488 | 0.488 | 27.935 | 0.076 | 0.073 | 8.407 |  |  |  |
|  | 0.488 |  |  | 0.07 |  |  |  |  |  |
| **Vehicle** | 0.364 | 0.363 | 18.372 | 0.086 | 0.085 | 11.172 |  |  |  |
|  | 0.362 |  |  | 0.084 |  |  |  |  |  |
|  | 0.319 | 0.322 | 15.294 | 0.08 | 0.082 | 10.480 |  |  |  |
|  | 0.325 |  |  | 0.084 |  |  |  |  |  |
| **Solution-3** | 0.526 | 0.533 | 31.446 | 0.083 | 0.083 | 10.711 |  |  |  |
|  | 0.54 |  |  | 0.083 |  |  |  |  |  |
|  | 0.466 | 0.469 | 26.463 | 0.078 | 0.079 | 9.789 |  |  |  |
|  | 0.472 |  |  | 0.08 |  |  |  |  |  |

Additional file 1 - References

1. Tang, D., et al., *SRplot: A free online platform for data visualization and graphing.* PLoS One, 2023. **18**(11): p. e0294236.

2. Breuer, K., et al., *InnateDB: systems biology of innate immunity and beyond--recent updates and continuing curation.* Nucleic Acids Res, 2013. **41**(Database issue): p. D1228-33.
